# Formic Acid‐Assisted Selective Hydrogenolysis of 5‐Hydroxymethylfurfural to 2,5‐Dimethylfuran over Bifunctional Pd Nanoparticles Supported on N‐Doped Mesoporous Carbon

**DOI:** 10.1002/anie.202012816

**Published:** 2021-01-22

**Authors:** Bin Hu, Lisa Warczinski, Xiaoyu Li, Mohong Lu, Johannes Bitzer, Markus Heidelmann, Till Eckhard, Qi Fu, Jonas Schulwitz, Mariia Merko, Mingshi Li, Wolfgang Kleist, Christof Hättig, Martin Muhler, Baoxiang Peng

**Affiliations:** ^1^ Laboratory of Industrial Chemistry Ruhr-University Bochum 44780 Bochum Germany; ^2^ Max Planck Institute for Chemical Energy Conversion 45470 Mülheim a.d. Ruhr Germany; ^3^ Chair of Theoretical Chemistry Ruhr-University Bochum 44780 Bochum Germany; ^4^ School of Petrochemical Engineering Changzhou University China; ^5^ Interdisciplinary Center for Analytics on the Nanoscale University of Duisburg-Essen 47057 Duisburg Germany

**Keywords:** DFT, formic acid, HMF, hydrogenolysis, metal-support interactions, Pd

## Abstract

Biomass‐derived 5‐hydroxymethylfurfural (HMF) is regarded as one of the most promising platform chemicals to produce 2,5‐dimethylfuran (DMF) as a potential liquid transportation fuel. Pd nanoparticles supported on N‐containing and N‐free mesoporous carbon materials were prepared, characterized, and applied in the hydrogenolysis of HMF to DMF under mild reaction conditions. Quantitative conversion of HMF to DMF was achieved in the presence of formic acid (FA) and H_2_ over Pd/NMC within 2 h. The reaction mechanism, especially the multiple roles of FA, was explored through a detailed comparative study by varying hydrogen source, additive, and substrate as well as by applying in situ ATR‐IR spectroscopy. The major role of FA is to shift the dominant reaction pathway from the hydrogenation of the aldehyde group to the hydrogenolysis of the hydroxymethyl group via the protonation by FA at the C‐OH group, lowering the activation barrier of the C−O bond cleavage and thus significantly enhancing the reaction rate. XPS results and DFT calculations revealed that Pd^2+^ species interacting with pyridine‐like N atoms significantly enhance the selective hydrogenolysis of the C−OH bond in the presence of FA due to their high ability for the activation of FA and the stabilization of H^−^.

## Graphical abstract

Quantitative conversion of HMF to DMF was achieved in the presence of formic acid and H_2_ over the bifunctional Pd/NMC catalyst within 2 h. Experimental investigation and DFT calculation revealed that formic acid significantly enhances the reaction rate and shifts the dominant reaction pathway from the hydrogenation of the aldehyde group to the hydrogenolysis of the hydroxymethyl group via its protonation.

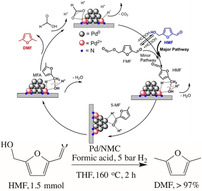

## Introduction

The utilization of renewable biomass feedstocks for the production of biofuels and commodity chemicals has attracted significant attention.[^1^, ^2^] 5‐Hydroxymethylfurfural (HMF) is regarded as one of the most important platform chemicals during biomass transformation.[^3^, ^4^] Various chemicals and biofuels, such as levulinic acid,^[5]^ 2,5‐dimethylfuran,^[6]^ 2,5‐furandicarboxylic acid,^[7]^ and ethyl levulinate,^[8]^ can be produced from HMF. The selective hydrogenolysis of HMF to DMF is a promising utilization route for obtaining biofuel from renewable feedstock.^[9]^ DMF is considered as a potential liquid transportation biofuel, which has a higher octane number than ethanol, superior energy intensity, nearly ideal boiling point, excellent miscibility with gasoline and immiscibility with water.^[10]^ Moreover, DMF is also a renewable source for the production of *p*‐xylene via the Diels–Alder reaction.^[11]^

**Scheme 1:**
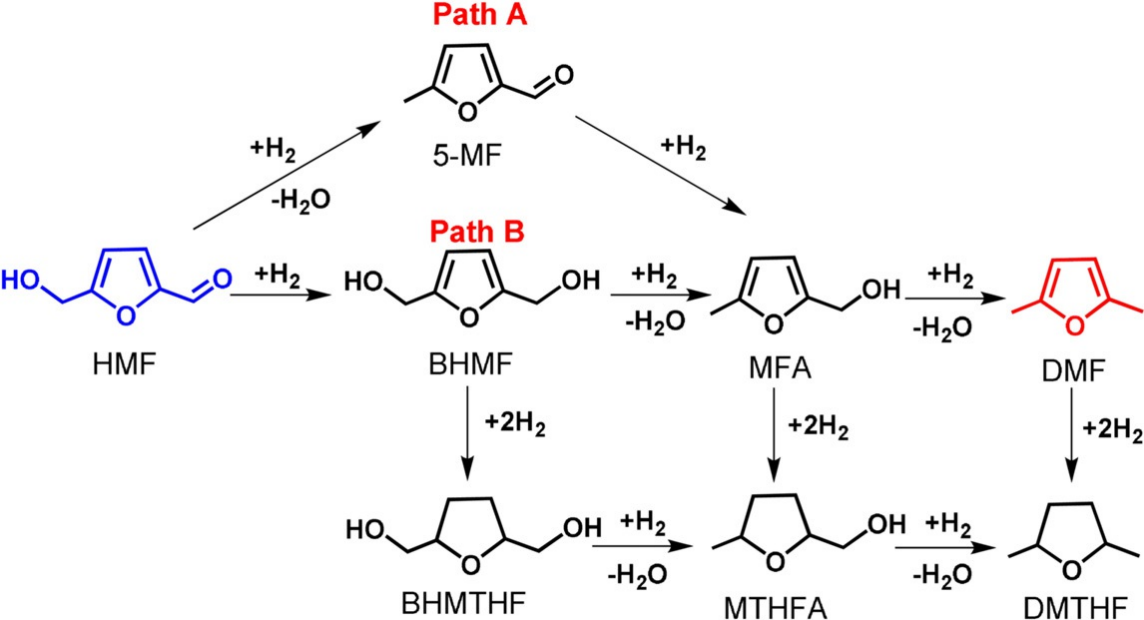
Reaction network for the hydrogenolysis of HMF to DMF.

The common route to produce DMF is the hydrogenolysis of carbohydrates or HMF with hydrogen, for which a large variety of catalysts has been investigated. In 2007, Dumesic et al. reported a two‐step catalytic process, resulting in 71 % yield of DMF from fructose via HMF as intermediate over Cu/C at 220 °C and 6.8 bar H_2_.[^6^, ^12^] In 2010, Bell et al. reported a two‐step approach for the conversion of glucose to DMF, achieving 15 % DMF yield based on Pd/C in ionic liquid under high pressure of H_2_ (62 bar).^[13]^ Recently, Abu‐Omar et al. synthesized a bimetallic catalyst containing Lewis‐acidic Zn and Pd/C species and applied it in the hydrogenolysis of HMF to DMF, leading to 99 % conversion and 85 % DMF selectivity, but the bimetallic catalyst suffered from deactivation after the 4th recycle.^[14]^ Yang et al. reported that Ru‐MoO_*x*_/C led to a DMF yield of 79 % at 180 °C and 15 bar H_2_.^[15]^ Generally, relative harsh reaction conditions, especially high pressures of H_2_, were employed to obtain appropriate conversion due to the low solubility of H_2_.^[16]^ Another key challenge for upgrading HMF is to obtain high product selectivity. The chemical transformation of HMF to DMF is a multistep process, including the hydrogenolysis of the hydroxymethyl group and the hydrogenation of the aldehyde group (Scheme 1). Importantly, the main side reaction of furan‐ring hydrogenation should be avoided. Overall, the highly selective full conversion of HMF to DMF under mild reaction conditions is still a challenge.

Molecular hydrogen is widely employed in hydrogenation/hydrogenolysis processes, owing to its high availability and facile activation on metal surfaces. Formic acid (FA), which can be produced from lignocellulosic biomass or electrochemical reduction of CO_2_, is being considered for reversible hydrogen storage as well as a hydrogen donor for catalytic hydrogenation.^[17]^ Compared with H_2_, FA not only has a higher energy density of 6.4 MJ L^−1^,^[18]^ but also has excellent stability and low toxicity, which is desirable for safe storage and transportation.^[19]^ Rauchfuss et al. reported the conversion of HMF with FA under mild conditions over Pd/C, yielding 95 % DMF in 15 h.[^20^, ^21^] The key strategy is the utilization of FA, which serves multiple roles as acidic catalyst, hydrogen donor, catalyst moderator inhibiting decarbonylation and ring hydrogenation, and as precursor to formate esters, which are key intermediates that are activated toward the selective hydrogenolysis of the C−OH bond. Tao et al. also reported a DMF yield of 98 % from HMF and FA over 1 % Au^Pd_0.2_/t‐ZrO_2_.^[22]^ Nevertheless, the different roles of FA have not yet been elucidated in detail.

Pd exhibits high catalytic activity in the hydrogenolysis of HMF to DMF using FA as the hydrogen source, and it is usually supposed to be the optimum metal for the decomposition of FA.^[23]^ N‐doped carbon materials have been proven to be promising supports.^[24]^ Doping with electron‐rich N atoms modifies the surface structure of carbon materials and endows improved π‐binding ability and enhanced basicity,^[25]^ which enhances the catalytic activity of supported metal catalysts.[^26^, ^27^, ^28^] For example, isolated Pd^2+^ cations supported on N‐doped carbon were identified as active sites for the H_2_ production from FA, which showed an enhanced reaction rate of 2–3 times compared with Pd supported on N‐free carbon.^[29]^ It was also demonstrated that the Pd^2+^ species are coordinated by pyridinic N according to XPS, NEXAFS, and STEM studies.^[29]^ Arrigo et al. reported a detailed study of metal‐support interactions of N‐doped carbon nanotubes with Pd^2+^ species.^[30]^ In our previous study, we were able to prove the presence of bifunctional Pd nanoparticles (NPs) supported on N‐doped mesoporous carbon (NMC), containing both Pd^0^ and Pd^2+^ active sites.^[31]^

Our present work aims at developing the quantitative conversion of HMF to DMF under mild reaction conditions, obtaining a comprehensive understanding of the versatile roles of FA, and revealing the influence of the bifunctionality of the Pd NPs on the reaction. Thus, a systematic comparative study was performed by variation of the catalyst support (N‐rich vs. N‐free mesoporous carbon (CMC)) and the hydrogen donor (H_2_, FA, both H_2_ and FA). In situ ATR‐IR spectroscopy was employed to monitor the reaction progress and to explore the reaction pathways. Density functional theory (DFT) was applied to gain further insight into the bifunctional electronic properties of Pd/NMC and its interaction with FA.

## Results and Discussion

### Characterization

The actual Pd loadings of the catalysts determined by AAS were found to be ca. 1.0 wt % close to their expected values (Table S1). The isotherms can be classified as type IV with H3 hysteresis loops for CMC and NMC representing typical mesopores (Figure S1). CMC displays a large specific surface area of 997 m^2^ g^−1^, while the surface area of NMC is smaller but still exhibits a surface area of 657 m^2^ g^−1^ (Table S1). The similar pore volumes and average pore sizes of these two carbon materials clearly indicate comparable mesoporous structures. After the colloidal deposition of Pd, the BET surface areas, pore sizes, and pore volumes of both supported Pd catalysts slightly decreased compared with those of the parent materials. The properties of active carbon (AC) and Pd/AC are also listed in Table S1.

XRD patterns show that all samples exhibit the characteristic reflections of graphitic carbon at ca. 26° (Figure S2). Pd/CMC shows clearly visible Pd(111) reflections at 40.2°, suggesting the presence of a larger number of Pd NPs above 5 nm, which can be detected by XRD.[^32^, ^33^] By comparison, no significant Pd reflections are identified in Pd/NMC, implying a higher Pd dispersion and that most Pd NPs are smaller than the XRD detection limit of about 5 nm. This result points to the crucial role of N atoms in NMC as strong anchoring sites for Pd NPs. The observation is in good agreement with previous reports, showing that N‐doped carbon and TiO_2_ can effectively anchor metal NPs and enhance their dispersion.[^34^, ^35^]

**Figure 1:**
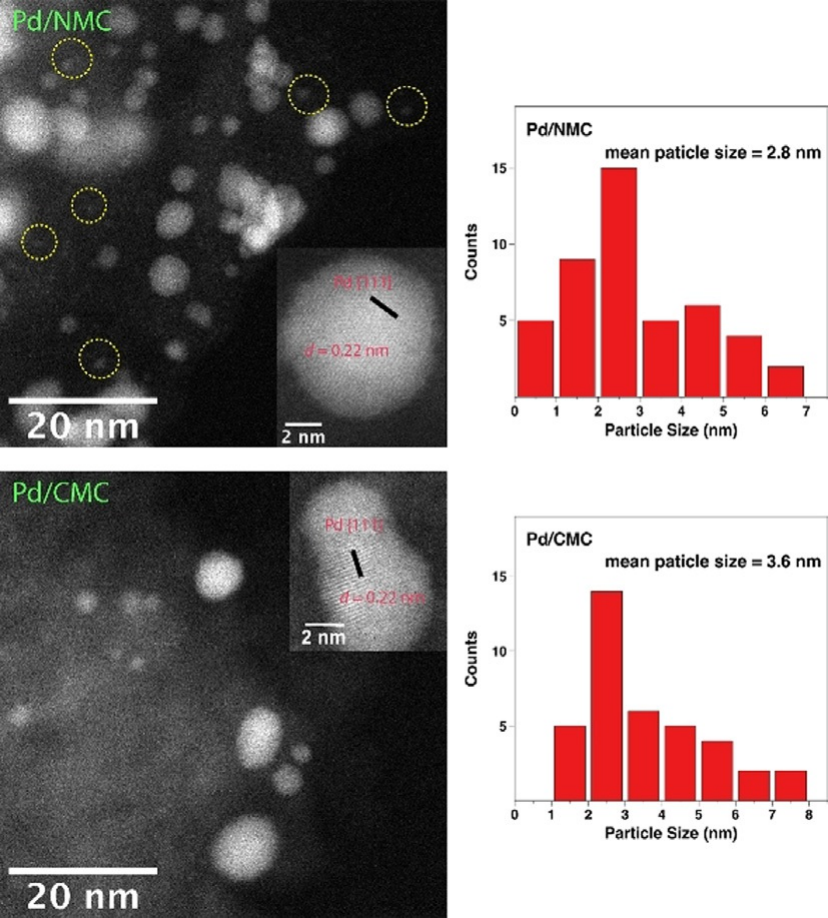
HAADF‐STEM images and Pd particle size distributions for Pd/NMC and Pd/CMC. The yellow circles refer to sub‐1 nm Pd NPs or clusters.

Pd NPs in the range of 2 to 5 nm can be clearly seen in the TEM images of both Pd/NMC and Pd/CMC (Figure S3). The mean Pd particle sizes for Pd/NMC and Pd/CMC are relatively similar amounting to 2.8 and 3.6 nm, respectively. Nevertheless, the HAADF‐STEM images show that there are some sub‐1 nm Pd NPs or clusters in Pd/NMC, while these sub‐1 nm NPs are rarely detected in Pd/CMC (Figure 1). The presence of sub‐1 nm NPs points to fragmentation occurring during the colloidal deposition. The lattice fringe spacings of both catalysts are determined to be 0.22 nm representing Pd(111) lattice planes. By comparison, Pd/AC has a larger mean Pd particle size of 4.4 nm as well as a significantly larger number of NPs above 5 nm (Figure S4).

**Figure 2:**
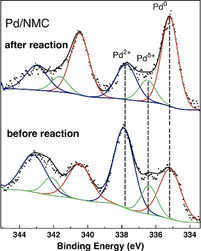
Pd 3d regions of the deconvoluted XPS results for Pd/NMC before and after reaction in the presence of FA and H_2_.

XPS results reveal that the surface Pd concentration of Pd/NMC (0.61 at %) (Figure 2) is much lower than that of Pd/CMC (1.83 at %) (Table 1, Figure S6). As both catalysts have similar bulk Pd loadings and Pd/NMC has smaller particle size and better dispersion than Pd/CMC according to the STEM measurements, the significant difference in the surface Pd concentration implies that Pd is presumably homogeneously dispersed both on the outer and inner surface of NMC due to the incorporation of N atoms, while Pd prefers to interact with the O‐functionalized groups and deposits on the outer CMC surface. Furthermore, the N 1s core level XP spectra of NMC demonstrate the presence of pyridinic (45 %), pyrrolic (46 %), quaternary‐like (6 %), and oxidized or NO‐like N atoms (3 %) in NMC (Figure S7).^[31]^ For Pd/NMC, a better fitting of the N 1s spectrum was obtained when assuming the presence of another N species interacting with Pd NPs (N‐Pd component).

**Table 1:** Chemical compositions of NMC, CMC, Pd/NMC, and Pd/CMC.

Sample	Pd [wt/at %]			N [wt/at %]			O [at %]
	bulk^[a]^	surf.^[b]^		bulk^[c]^	surf.^[b]^		surf.^[b]^
NMC	–	–		13.4	10.6		6.3
CMC	–	–		–	–		11.0
Pd/NMC	0.95	0.61		13.3	10.9		7.4
Pd/CMC	0.98	1.83		–	–		11.5

Determined by [a] AAS, [b] XPS and [c] elemental analysis.

**Figure 3:**
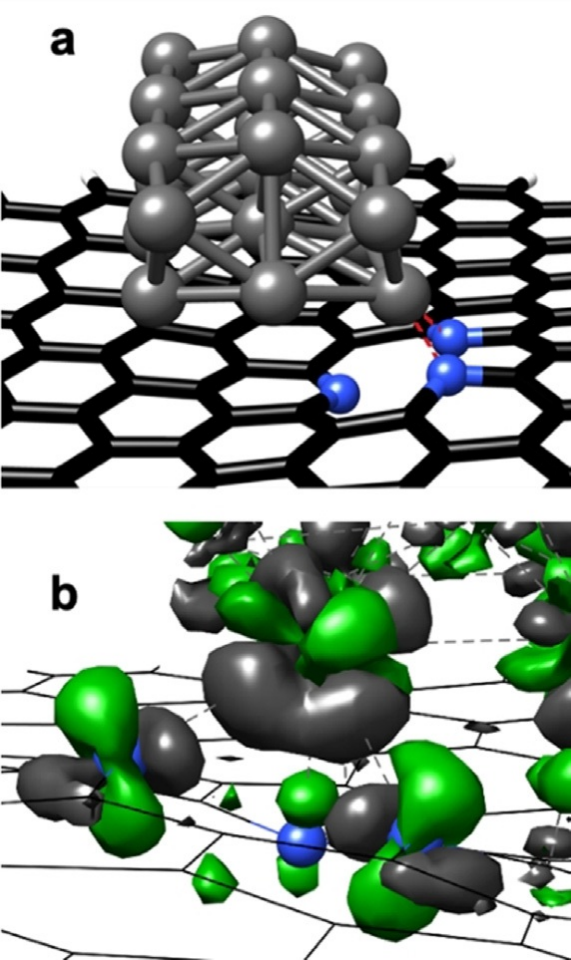
DFT calculations for Pd/NMC model: (a) most stable structure of Pd_21_ on pyridinic carbon support (two pyridinic N connected to Pd_21_); (b) change in the electron density induced by the interaction between the Pd_21_ cluster and the pyridinic N atoms (green: density gain; grey: density loss).

In our previous studies, we were able to achieve an in‐depth insight into the metal‐support interactions of Pd/CMC and Pd/NMC.[^31^, ^36^] Briefly, Pd NPs interact via directed and partially covalent Pd‐C interactions with the carbon support.^[36]^ Figure 3 a shows the most stable structure of a Pd_21_ cluster with a calculated diameter of 0.85 nm on NMC determined by ground‐state geometry optimization. Two pyridinic N atoms are interacting with one Pd atom, suggesting pyridinic N atoms as the preferential adsorption sites for Pd clusters. The relevant part of the corresponding electron density difference is reproduced in Figure 3 b. The complicated structure can be explained with the help of an intrinsic bond orbital analysis, showing that the pyridinic N atoms possess a lone pair (LP), located in a sp^2^‐orbital lying in the support plane. When a Pd cluster is adsorbed on pyridinic N, two N atoms are interacting with one Pd atom via an N‐LP—Pd d_σ_ and an N‐LP—Pd s as well as a Pd d_π_–π* charge transfer. The interaction of the N‐LP orbitals with the Pd atom leads to a density loss in the N‐LP orbital, while the Pd d_π_–π* interaction increases the density in the N π orbital and leads to a change in the Pd oxidation state to Pd^2+^. Indeed, only Pd^0^ and Pd^δ+^ are observed by XPS for Pd/CMC (Figure S8), whereas an additional peak, which can be attributed to a divalent Pd species stabilized by the strong interaction with pyridine‐like N atoms,^[30]^ is observed for Pd/NMC (Figure 2). Pd^0^ (68 %) is the dominant species for Pd/CMC, while Pd^2+^ (50 %) is the main species for Pd/NMC (Table S2). These results prove the presence of bifunctional Pd NPs containing both Pd^0^ and Pd^2+^ active sites, supported by pyridinic N atoms in NMC.^[31]^ In addition, XAS results of as‐prepared samples also strongly support the structure model of small Pd clusters containing oxidized Pd^δ+^ or Pd^2+^ centers at their edges, which are attached to the support via O or N atoms (see detailed XAS and TPR analysis in Supporting Information). The higher oxidation state of Pd in Pd/NMC compared to Pd/CMC is also verified by TPR experiments.

### Catalytic Conversion of HMF to DMF

The conversion of HMF can proceed via two pathways, depending on the sequence of hydrogenolysis of the hydroxymethyl group and hydrogenation of the aldehyde group (Scheme 1). These two pathways produce distinct intermediates: 5‐methylfurfural (5‐MF) for path A and 2,5‐bis(hydroxymethyl)furan (BHMF) for path B. Both paths proceed further to a common intermediate 2‐(hydroxymethyl)‐5‐methylfuran (MFA) followed by subsequent hydrogenolysis to DMF. The hydrogenation of the furan ring leads to undesired byproducts. The hydrogenolysis of HMF to DMF with different hydrogen sources over Pd‐based catalysts was investigated and summarized in Table 2.

**Table 2:** Product distributions of HMF hydrogenolysis over the supported Pd catalysts.^[a]^

Entry	Catalysts	Hydrogen Source	Conversion [%]	DMF Selectivity [%]	Yields [%]				
					DMF	BHMF	5‐MF	MFA	Others^[e]^
1	–	H_2_^[b]^	4.6	–	–	2.2	2.4	–	–
2	Pd/NMC	H_2_^[b]^	>99.9	35.3	35.3	–	–	42.0	22.7
3	Pd/CMC	H_2_^[b]^	78.8	49.2	38.8	–	6.6	18.3	15.1
4	Pd/AC	H_2_^[b]^	19.4	38.3	7.4	1.9	8.4	0.6	1.0
5	Pd/NMC	FA^[c]^	60.8	64.3	39.1	–	11.5	10.2	–
6	Pd/CMC	FA^[c]^	21.4	62.9	13.5	–	7.9	–	–
7	Pd/NMC	FA+H_2_^[d]^	>99.9	>97.0	97.1	–	2.9	–	–
8	Pd/CMC	FA+H_2_^[d]^	90.1	80.0	72.1	–	18.0	–	–

[a] Reaction conditions: 1.5 mmol HMF, 50 mg catalysts, 160 °C, 3 h; [b] 5 bar H_2_; [c] 45 mmol FA (30 equiv) at 5 bar N_2_ and [d] H_2_; [e] mainly ring‐hydrogenated products including 2,5‐bis(hydroxymethyl)tetrahydrofuran, 5‐methyltetrahydrofurfuryl alcohol, and 2,5‐dimethyltetrahydrofuran.

**Figure 4:**
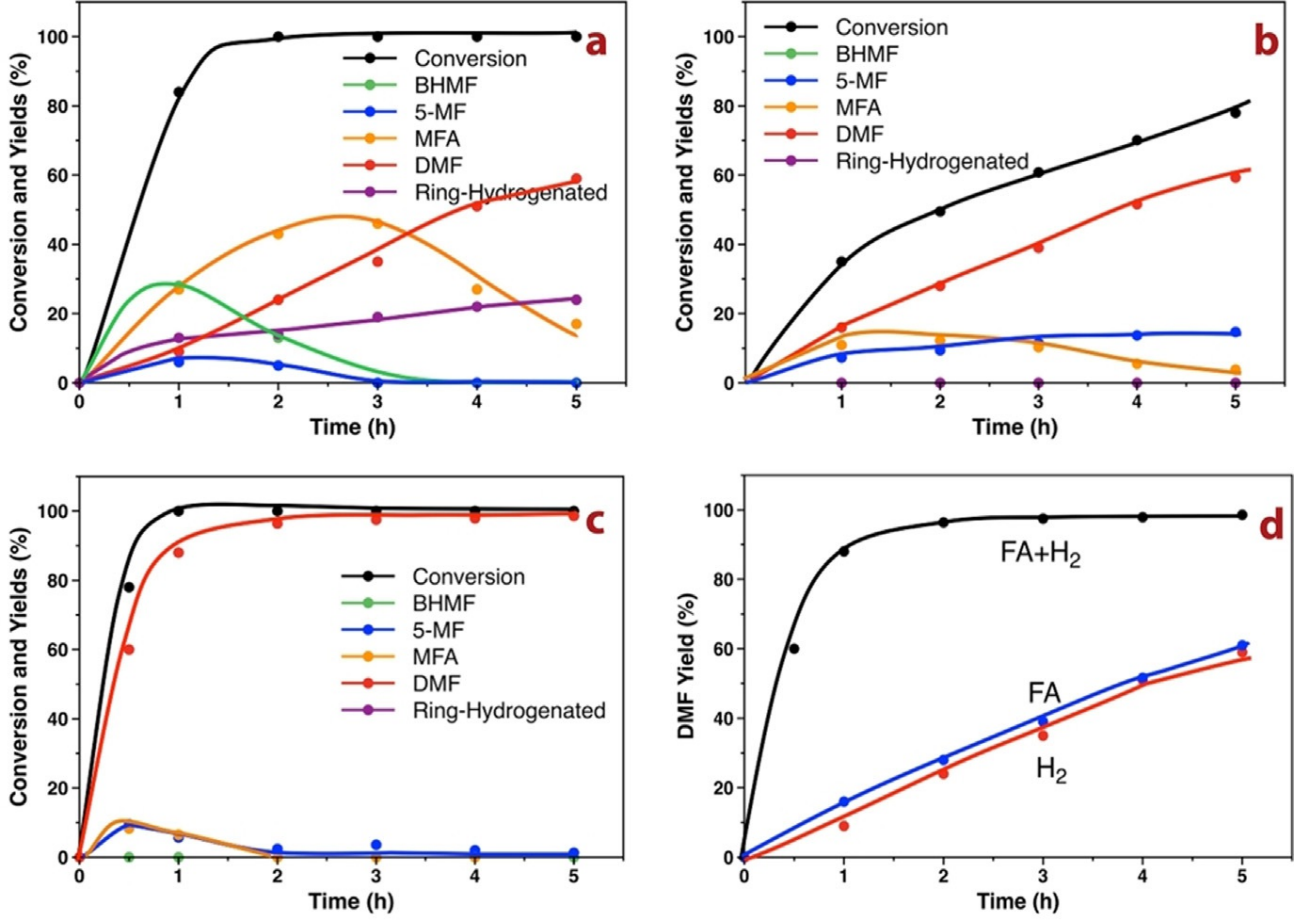
Time course of the hydrogenolysis of HMF to DMF over Pd/NMC with (a) 5 bar H_2_; (b) FA and 5 bar N_2_; (c) FA and 5 bar H_2_. (d) Comparison of DMF yields using different hydrogen donors over time.

When H_2_ was used, the blank experiment led to only 4.6 % conversion, and DMF was not observed (Table 2, entry 1). The experiments with NMC and CMC resulted in similar conversion as the blank experiment. The degrees of HMF conversion over the Pd‐based catalysts were 99.9 %, 78.8 %, and 19.4 % for Pd/NMC, Pd/CMC, and Pd/AC (Table 2, entries 2–4), respectively. The yields of DMF were found to be similar for the mesoporous carbon‐supported Pd catalysts, amounting to 35.3 % for Pd/NMC and 38.8 % for Pd/CMC, while the yield was significantly lower for Pd/AC. The poor catalytic activity of Pd/AC is due to the larger Pd particle size and the lower Pd dispersion. Besides, mainly furan‐ring‐hydrogenation products were obtained as byproducts. The hydrogenolysis of HMF over Pd/NMC as a function of time was further studied (Figure 4 a). The conversion of HMF proceeded very fast, obtaining full conversion within 2 h. BHMF and 5‐MF were the primary intermediates, which reached a maximum yield within 1 h and then gradually decreased to zero after 4 h. By comparison, the maximum yield of BHMF was much higher than that of 5‐MF, suggesting that the BHMF reaction pathway (path B) excels the 5‐MF pathway (path A) for HMF hydrogenolysis in H_2_. MFA was found to be the secondary intermediate, which reached a maximum yield of 42 % at 3 h and then gradually decreased to 17.8 % accompanied by an increase in DMF yield to 59.6 % after 5 h. Additionally, the ring‐hydrogenation products continuously increased to 20 %, which is commonly observed in H_2_.[^37^, ^38^] By comparison, the product evolution profiles over Pd/CMC are rather similar to that over Pd/NMC, but with slightly lower activity (Figure S13).

When FA was used as a hydrogen donor, the degrees of conversion and product distributions changed significantly for both Pd/NMC and Pd/CMC (Table 2, entries 5, 6). The degrees of conversion decreased strongly, and the selectivities to DMF increased by 13–29 %. Ultimately, it resulted in a similar DMF yield (39 % vs. 35 %) over Pd/NMC after 3 h compared to that in H_2_ atmosphere. Notably, the hydrogenation of the furan ring, which commonly occurs and is difficult to avoid with H_2_, is completely suppressed in the presence of FA. This can be explained by the sterically hindered adsorption of the furan ring, because the active Pd sites are preferably covered by the more strongly bound formate anions. In contrast to the reaction in H_2_, the reaction progress in FA over Pd/NMC shows that BHMF was not detected, while only 5‐MF with similar concentration (10 %) was observed (Figure 4 b). This result suggests that the hydrogenolysis of the hydroxymethyl group (path A) precedes the hydrogenation of the aldehyde group (path B) in FA. After 5 h, a conversion of 78.1 % and a DMF yield of 60 % were obtained. By extending the reaction time to 12 h, the DMF yield increased to almost 90 %. It is worth noting that the yield of the primary intermediate 5‐MF slowly but continuously increased, while the secondary intermediate MFA first reached a plateau and then gradually decreased after 3 h. This observation suggests that the hydrogenation of 5‐MF to MFA is the rate‐determining step (rds) because of the relatively poor hydrogenation ability of Pd/NMC in FA.

**Scheme 2:**
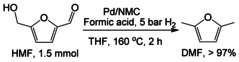
Quantitative conversion of HMF to DMF with FA and H_2_ over Pd/NMC.

In order to enhance the reaction rate of the rds, the conversion of HMF with FA was also conducted in the presence of additional external H_2_. The yields of DMF over Pd/NMC and Pd/CMC increased strongly to 97.5 % and 73.9 % (Table 2, entries 7, 8), respectively. Similar to the reaction progress in FA, Figure 4 c shows that BHMF was not detected and path A (5‐MF) is the main reaction pathway in the presence of external H_2_. The difference is that the change of MFA yield occurred concurrently with that of 5‐MF, indicating that the reaction rate of the hydrogenation of 5‐MF to MFA was significantly enhanced. Finally, the quantitative conversion of HMF to DMF is achieved within 2 h over Pd/NMC (Scheme 2). Compared with Pd/CMC, Pd/NMC exhibits superior catalytic activity with doubled TOF values both in FA and in H_2_+FA (Table S4), suggesting strong promoting effects of FA and the N species in Pd/NMC. Furthermore, Pd/NMC outperforms other reported catalysts in terms of TOF (Table S4), which can be attributed to smaller Pd NPs, strong metal‐support interactions and modified electronic properties by the N species.

By comparison, the combination of FA and H_2_ strongly enhances the hydrogenolysis of HMF to DMF, obtaining excellent HMF conversion and DMF yield in short reaction time (Figure 4 d). The addition of FA changes the reaction pathway from path B to path A, enhances the hydrogenolysis ability, and inhibits the ring‐hydrogenation, whereas the presence of H_2_ ensures the fast hydrogenation step.

**Figure 5:**
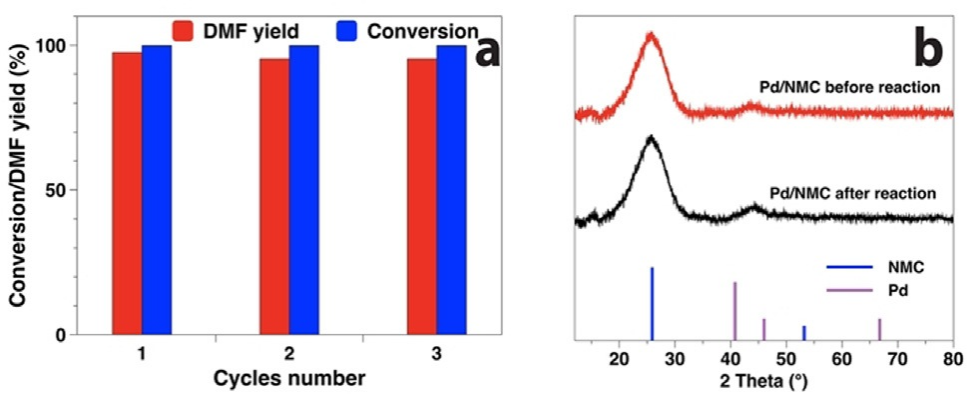
Reusability of Pd/NMC in the hydrogenolysis of HMF to DMF in the presence of FA and H_2_ (a), and XRD patterns of Pd/NMC before and after reaction (b).

The stability and the reusability of Pd/NMC were also evaluated. The used catalyst kept its high catalytic activity after three runs, and the yield of DMF only slightly decreased from 97.5 % to 95.1 % (Figure 5 a). The low degree of Pd leaching, which is only 0.06 % (0.9 ppm) of the total Pd, indicates an efficient stabilization of the Pd NPs by the NMC support. Furthermore, the recycled catalyst was characterized by XRD, TEM, and XPS (Figures 5 b, S5, and 2). The Pd(111) reflection in the used catalyst is still hardly visible without significant changes as compared to the fresh catalyst. The mean Pd particle size increased slightly from 2.8 to 2.9 nm and the sub‐1 nm Pd NPs are still visible. After reaction, the relative abundance of Pd^0^ in Pd/NMC increased from 36 to 59 %, while Pd^2+^ decreased from 50 to 30 % (Table S2). For comparison, Pd/CMC was almost completely reduced to Pd^0^ (91 %) after reaction. The moderate reduction of Pd/NMC even in the presence of FA and H_2_ is ascribed to the strong anchoring by pyridinic N species. Overall, Pd/NMC is highly stable and exhibits excellent reusability.

### Reaction Mechanism

**Figure 6:**
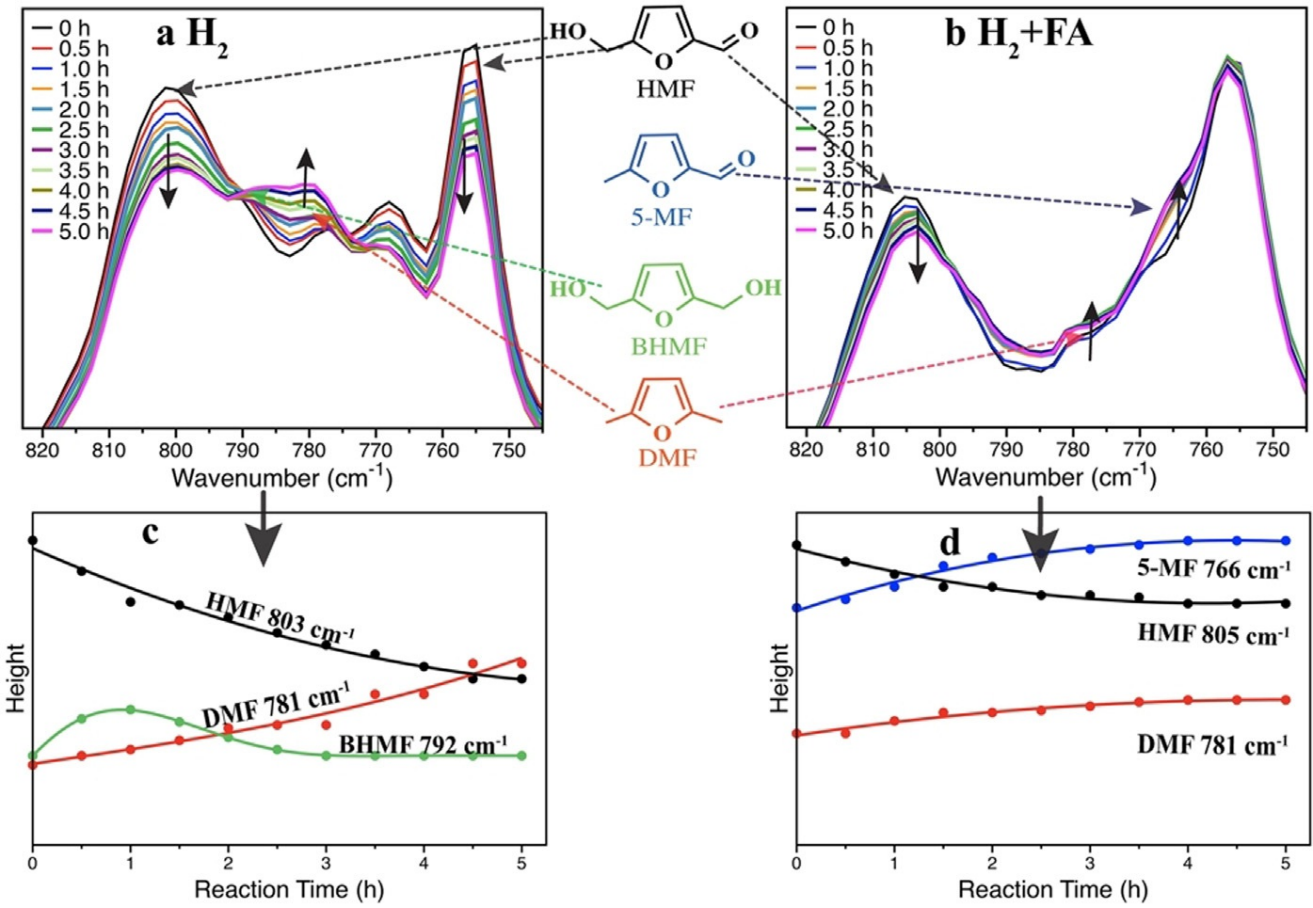
In situ ATR‐IR spectra and qualitative trends of peak heights during HMF conversion over Pd/NMC in (a) (c) 10 bar H_2_ after subtraction of the background of THF; (b) (d) 15 mL FA at 10 bar H_2_ after subtraction of the background of THF and FA. Reaction conditions: 45 mmol HMF, 500 mg catalysts, 120 mL THF, 10 bar H_2_, 160 °C, 500 rpm.

In situ ATR‐IR spectroscopy was employed to investigate the reaction pathway and product distributions with different hydrogen donors. Figure 6 a and 6 b depict the time‐resolved ATR‐IR spectra collected during the hydrogenolysis of HMF to DMF over Pd/NMC at 160 °C by H_2_ and by H_2_+FA, respectively. The reference spectra of the main compounds are shown in Figure S14.

The assignment of the characteristic bands to the reactant and intermediates is summarized in Table S5. Due to the similarity of the intermediates and the reactant, the differentiation of C=O and C=C stretching vibrations is impossible, and therefore, the bands of out‐of‐plane C−H bending were compared in detail, using the characteristic C−H bending vibrations for HMF (805, 779, 767 and 756 cm^−1^), DMF (781 cm^−1^), 5‐MF (796 and 765 cm^−1^), and BHMF (792 cm^−1^).

Using H_2_ as the hydrogen donor, the intensity of the IR bands at 805 and 756 cm^−1^ (HMF) continuously declined, whereas the intensity of the IR band at 781 cm^−1^ (DMF) gradually increased. The IR peak of 5‐MF was not detected probably due to the low concentration, while the peak at 792 cm^−1^ (BHMF) first formed and then declined. The time‐resolved evolution of these peaks is presented in Figure 6 c, which once again verifies the conclusion that Pd/NMC tends to catalyze the hydrogenation of the aldehyde group (path B) compared with the hydrogenolysis of the hydroxymethyl group (path A) in H_2_.

In the case of FA and H_2_ as hydrogen donors, the consumption of HMF and the formation of DMF were still observed (Figure 6 b). Nevertheless, the intensities of the IR bands at 765 and 796 cm^−1^ (5‐MF) increased significantly without the formation of BHMF in good agreement with the GC results (Table 2, Figure 4). These bands were deconvoluted and plotted in Figure 6 d. The decrease of HMF as well as the increase of DMF and the primary intermediate 5‐MF (path A) can be clearly observed in the presence of FA+H_2_.

With respect to the mechanism, FA not only changes the dominant reaction pathway from path B to path A, but also significantly improves the conversion of HMF to DMF over Pd/NMC, which is proposed to proceed via the enhancement of the hydrogenolysis of the C−OH bond (path A). On the contrary, path B was identified as the main reaction pathway over Pd/C in the presence of FA and H_2_ in a previous report,^[21]^ implying that Pd/NMC may also play a role in the shift of reaction pathway.

FA is considered to serve multiple roles: it acts as a mild hydrogen source, a catalyst moderator inhibiting ring hydrogenation, and a precursor to formate esters as key intermediates that are activated toward selective hydrogenolysis of C−OH bonds.[^20^, ^21^] The role of FA as a hydrogen source in the absence of external H_2_ is rather clear. The suppression of ring‐hydrogenation resulting in improved selectivity has been clearly observed in the present study, but the differentiation or clarification of other roles affecting catalytic activity has not been investigated yet. Therefore, further experiments were conducted to probe the influence of FA on the hydrogenolysis and hydrogenation of furanic substrates catalyzed by Pd/NMC with additional external H_2_ (Table 3). When strongly decreasing the molar ratio of FA to HMF from 30 via 3 to 0.3, only a slight decrease of HMF conversion and DMF yield was observed (Table 3, entries 3–5), implying that FA only has a limited role as a hydrogen source in the presence of external H_2_. Furthermore, by addition of only 0.3 equiv FA, the yield of DMF increased significantly from 35.3 % to 76.0 %. Although ester formation was restricted using such a low concentration of FA, excellent DMF yield was still obtained. This result suggests that the formate ester pathway might not be the main route for HMF hydrogenolysis under the employed reaction conditions. In order to further verify the role of the formate ester, the conversion of 5‐[(formyloxy)methyl]furfural (FMF) to DMF was investigated (Table 3, entries 8–10). Compared with the conversion of HMF, lower degrees of conversion and DMF yields were obtained for all cases. Thus, it can be concluded that the reaction pathway of formate ester as an intermediate is a minor route for the hydrogenolysis of HMF to DMF. Note that FMF was not detected in all experiments, probably because the conversion of FMF to DMF is much faster than the formation of FMF under the present reaction conditions.

**Table 3:** Effect of additives on the conversion of HMF and FMF to DMF.^[a]^

Entry	Catalysts	Reactant	Additive^[b]^	Gas Phase	Conversion [%]	DMF Yield [%]
1	Pd/NMC	HMF	–	H_2_	>99.9	35.3
2	Pd/NMC	HMF	FA	N_2_	60.8	39.1
3	Pd/NMC	HMF	FA	H_2_	>99.9	>97.0
4	Pd/NMC	HMF	FA (3 equiv)	H_2_	92.0	89.3
5	Pd/NMC	HMF	FA (0.3 equiv)	H_2_	82.1	76.0
6	Pd/NMC	HMF	AcOH^[c]^	N_2_	10.6	Trace
7	Pd/NMC	HMF	AcOH^[c]^	H_2_	95.1	85.5
8	Pd/NMC	FMF	–	H_2_	66.1	46.0
9	Pd/NMC	FMF	FA	N_2_	34.2	20.5
10	Pd/NMC	FMF	FA	H_2_	83.1	67.3

[a] Reaction conditions: 1.5 mmol HMF or FMF, 50 mg catalysts, 160 °C, 5 bar H_2_ or N_2_, 3 h; [b] 45 mmol FA (30 equiv); [c] 45 mmol acetic acid (30 equiv).

Furthermore, the addition of acetic acid (p*K*_a_ 4.7 vs. 3.8 for FA) instead of FA led to only 10.6 % HMF conversion and traces of DMF (Table 3, entry 6), demonstrating that acetic acid cannot be used as the hydrogen donor. Nevertheless, excellent HMF conversion and DMF yield of 85.5 % were obtained in the presence of acetic acid and additional external H_2_ (Table 3, entry 7). In addition, 5‐MF was detected as primary intermediate and BHMF was not detected in good agreement with the result using FA. These results suggest that the acids probably enhance the hydrogenolysis of the RCH_2_−OH bond via an acid‐catalyzed route. Assary et al.^[39]^ proposed the reaction mechanism of fructose dehydration to HMF using high‐level quantum chemical methods. It was demonstrated that the acidity of the solution is essential to lower the activation energy barrier by involving initial protonation. Moreover, Lercher et al.^[40]^ reported a catalytic pathway for the cleavage of the C−O bond in benzyl phenyl ether. DFT calculations suggested that the ether is initially protonated rather than the direct cleavage of the ether bond in the proposed C−O bond cleavage mechanisms due to the lower activation energy. Combining our experimental observations and these literature reports, we propose that the hydrogenolysis of RCH_2_‐OH in the presence of acids also proceeds via a similar protonation process initially, which can lower the activation barrier of the C−O bond cleavage.

Overall, FA serves multiple roles in the hydrogenolysis of HMF to DMF: 1) suppressing ring‐hydrogenation to improve the DMF selectivity, 2) acting as a mild hydrogen source, 3) producing the formate esters as intermediates, 4) forming a protonated intermediate and thus lowering the activation barrier of the C−O bond cleavage. Functions 2–4 contribute together to the activity improvement, among which the protonation mechanism results in the reaction pathway shift from path B to path A, being the main reason for the significant catalytic activity enhancement.

**Figure 7:**
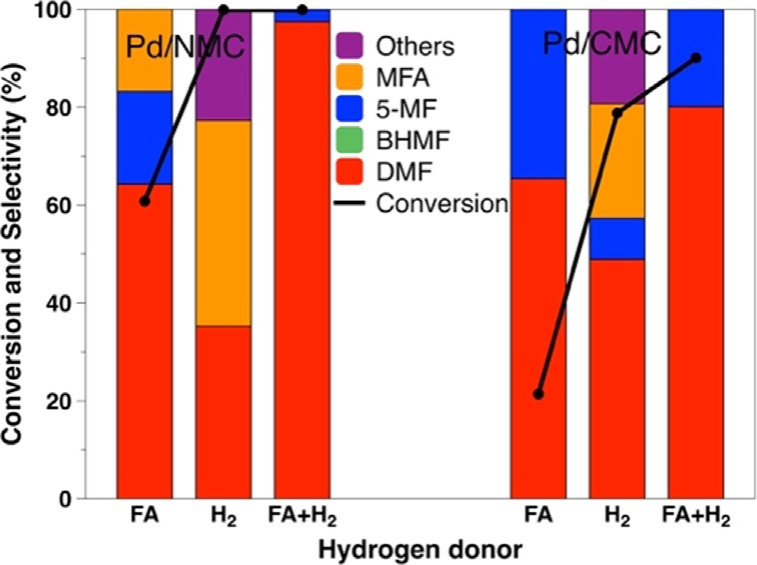
Comparison of Pd/NMC and Pd/CMC with respect to the conversion of HMF to DMF using different hydrogen sources after 3 h.

The catalytic performance of the hydrogenolysis of HMF over Pd/NMC and Pd/CMC with different hydrogen donors is presented in Figure 7. A significant difference in the reaction rate of the hydrogenolysis of the C−O bond was observed for N‐rich and N‐free carbon‐supported Pd catalysts. When H_2_ was employed, Pd/NMC and Pd/CMC led to similar DMF yields, implying their comparable catalytic activities for the hydrogenolysis of HMF in H_2_. Using FA as the hydrogen source, Pd/NMC resulted in both higher activity and DMF yield compared to Pd/CMC. In FA and H_2_, much higher conversion and yield over Pd/NMC were also obtained. As the main reaction pathway is the hydrogenolysis path A in the presence of FA or FA+H_2_, the higher catalytic activity of Pd/NMC can be attributed to the improved activity of the C−OH bond hydrogenolysis. Since Pd/NMC and Pd/CMC exhibit similar Pd particle sizes and mesoporous structures, the nature of the N atoms and different oxidation states of Pd species caused by strong metal‐support interactions should provide a clue.

**Figure 8:**
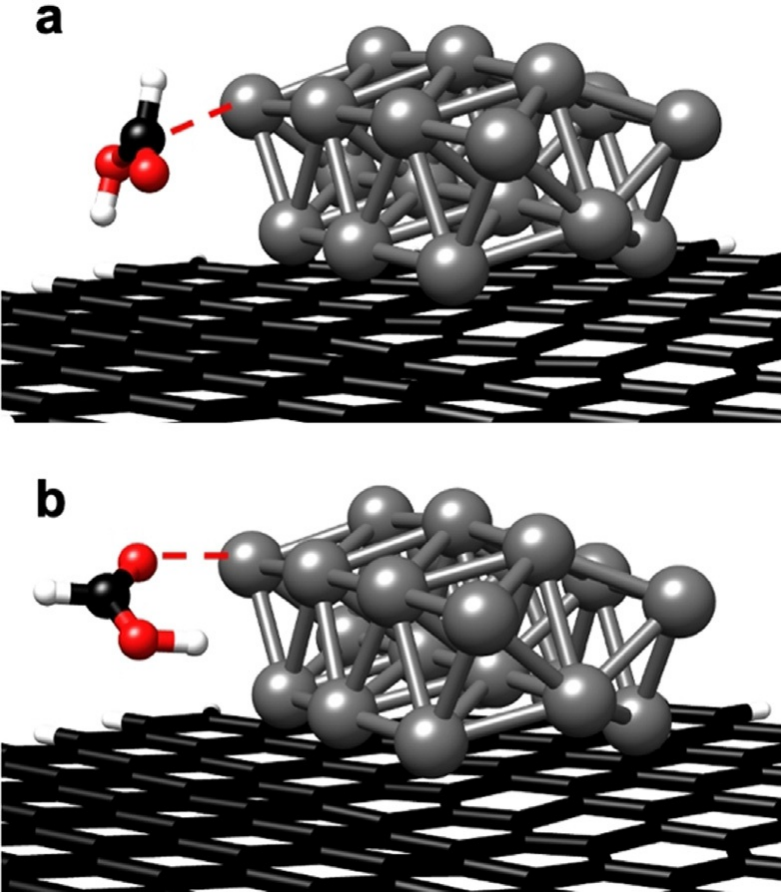
Two different binding configurations of FA on carbon supported Pd_21_ (model for Pd/CMC): (a) FA binds via the C atom; (b) FA binds via the C=O group.

To further identify the effect of FA and of the N‐doping of the support on the hydrogenolysis of the C−O bond, a DFT study of H_2_ and FA on Pd/CMC and Pd/NMC was performed (see detailed procedures and results in Supporting Information). The DFT calculations support the hypothesis that FA does not only act as a mild hydrogen source (function 2), but also can form protonated intermediates (function 4), as it interacts with carbon‐supported Pd NPs in two ways: a) FA binds via the C atom (Figure 8 a), subsequently decomposing into H_2_ and CO_2_; b) FA binds via the C=O group (Figure 8 b), subsequently decomposing into the formate anion and H^+^.

At the Pd_21_ cluster bound to NMC, we find that at the Pd^2+^ centers FA binds exclusively via the C=O bond and decomposes with a small activation energy (Δ*E*=5.3 kJ mol^−1^, neglecting solvation effects) into the formate anion and H^+^. The adsorption via the C atom as well as the reaction pathway to the formation of H_2_ and CO_2_ are unfavorable (Table S6). Accordingly, the bond to the positively charged center enhances the acidity of FA, which facilitates the hydrogenolysis of the C−OH bond of HMF. This is in agreement with previous studies, in which Pd^2+^ species were identified as the active sites for the selective decomposition of FA.^[29]^ For example, Pd^2+^ species supported on N‐rich carbon showed 3 times higher catalytic activity in FA decomposition compared with the activity of Pd supported on N‐free carbon.^[29]^ The isolated Pd^2+^ species stabilized by the pyridinic N atoms were identified as the active sites.

Furthermore, H^−^ formed by heterolytic H_2_ dissociation on the Pd nanoparticle binds significantly stronger to Pd/NMC compared with Pd/CMC, preferring positions close to the Pd^2+^ center (Table S7). This can be explained by the higher positive charge of Pd NPs on NMC, especially close to the Pd^2+^ center. The stabilization of H^−^ on Pd/NMC increases the nucleophilicity, and therefore, also the selectivity of H_2_ as a reducing agent.

In summary, the remarkable catalytic activity of Pd/NMC for HMF conversion in FA can be attributed to the shift to reaction path A and to the presence of Pd^2+^ species, showing higher catalytic activity in the FA dissociation to the formate anion and H^+^ as well as in the H^−^ stabilization, which jointly improve the hydrogenolysis of the C−OH bond significantly.

**Scheme 3:**
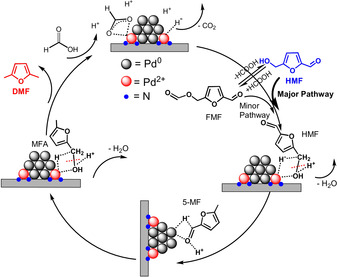
Proposed reaction pathway for the conversion of HMF to DMF over Pd/NMC in FA.

Based on the kinetic, spectroscopic and computational results, the following reaction pathway over the bifunctional Pd/NMC in FA is proposed (Scheme 3). First, FA is activated via Pd^2+^ species and Pd NPs. The reversible reaction between HMF and FMF exists in the presence of FA, but the HMF pathway is the main route instead of the formate ester pathway. Then, Pd^2+^ species bind the oxygen atom of the C‐OH group, which is protonated by FA. The hydrogenolysis of the hydroxymethyl group occurs via the cleavage of the C−OH bond forming 5‐MF. During the process, Pd^2+^ species and FA cooperate synergistically in the hydrogenolysis of the C−OH bond. Furthermore, Pd^0^ NPs bind the C=O double bond leading to the fast hydrogenation of 5‐MF to MFA. Subsequently, MFA is directly converted to DMF via a similar process like the hydrogenolysis of HMF to 5‐MF. In the case of FA and H_2_ as hydrogen donors, H_2_ is activated by the Pd^0^ NPs supported on NMC, which are more active for hydrogenation steps such as 5‐MF to MFA and HMF to BHMF. H_2_ may also directly contribute to the hydrogenolysis reactions, but probably plays a minor role in the presence of FA.

## Conclusion

Pd NPs supported on N‐doped and N‐free mesoporous carbon materials were successfully synthesized via the immobilization method and systematically characterized. These catalysts exhibit similar mean Pd particle sizes, but sub‐1 nm Pd clusters were only observed in Pd/NMC. DFT calculations and XPS measurements revealed the strong metal‐support interactions between Pd^2+^ species and pyridine‐like N atoms for Pd/NMC. The hydrogenolysis of HMF to DMF over the Pd‐based catalysts was investigated by varying different hydrogen sources. A favorable HMF full conversion, DMF yield of >97 % and a higher TOF of 150 h^−1^ were obtained over Pd/NMC in the presence of FA and H_2_ after 2 h.

Detailed comparative studies mainly by varying hydrogen source, additive, and reactant as well as by applying in situ ATR‐IR spectroscopy and DFT calculations were performed to explore the reaction mechanism. FA tunes not only the reactivity of HMF but also its reaction pathways, serving multiple roles, such as suppressing ring‐hydrogenation, acting as a mild reducing agent, producing the formate esters as intermediates, and forming a protonated intermediate, thus lowering the activation barrier of the C−OH bond cleavage. Protonation results in a reaction pathway shift from the hydrogenation of the C=O bond (path B) to the hydrogenolysis of the C−OH bond (path A) and is the main reason for the significant catalytic activity enhancement. Furthermore, a synergistic effect between FA and the Pd^2+^ species of Pd/NMC was observed for the hydrogenolysis of the C−OH bond, with Pd^2+^ exhibiting higher catalytic activity for the activation of FA and stabilization of H^−^.

## Conflict of interest

The authors declare no conflict of interest.

## Supporting information

As a service to our authors and readers, this journal provides supporting information supplied by the authors. Such materials are peer reviewed and may be re‐organized for online delivery, but are not copy‐edited or typeset. Technical support issues arising from supporting information (other than missing files) should be addressed to the authors.

Supplementary
