# Supplementary material for: Formic Acid‐Assisted Selective Hydrogenolysis of 5‐Hydroxymethylfurfural to 2,5‐Dimethylfuran over Bifunctional Pd Nanoparticles Supported on N‐Doped Mesoporous Carbon
Source: Angew Chem Int Ed Engl. 2021 Jan 22;60(12):6807–15. doi: 10.1002/anie.202012816 (PMC7986868; doi:10.1002/anie.202012816)
Supplement: Supplementary file 1 — Supplementary [file ANIE-60-6807-s001.pdf]

Angewandte Chemie
Eine Zeitschrift der Gesellschaft Deutscher Chemiker

Supporting Information

Formic Acid-Assisted Selective Hydrogenolysis of 5-Hydroxymethylfurfural to 2,5-Dimethylfuran over Bifunctional Pd Nanoparticles Supported on N-Doped Mesoporous Carbon

*Bin Hu, Lisa Warczinski, Xiaoyu Li, Mohong Lu, Johannes Bitzer, Markus Heidelmann, Till Eckhard, Qi Fu, Jonas Schulwitz, Mariia Merko, Mingshi Li, Wolfgang Kleist, Christof Hättig, Martin Muhler, and Baoxiang Peng**

anie_202012816_sm_miscellaneous_information.pdf

List of abbreviations

HMF	5-(Hydroxymethyl)furfural
LA	Levulinic acid
FDCA	2,5-Furan-dicarboxylic
EL	Ethyl levulinate
DMF	2,5-Dimethylfuran
DFF	2,5-Diformylfuran
FA	Formic acid
BHMF	2,5-Bis(hydroxymethyl)furan
5-MF	5-Methylfurfrual
MFA	2-(Hydroxymethyl)-5-methylfuran
DMTHF	2,5-Dimethyltetrahydrofuran
FMF	5-[(Formyloxy)methyl]furfural
BHMTHF	2,5-Bis(hydroxymethyl)tetrahydrofuran
MTHFA	5-Methyltetrahydrofurfuryl alcohol

Experimental

Materials

All commercially available reagents were used as received without further purification unless otherwise specified. Palladium chloride (99%), formic acid (97%), acetic acid (glacial), pyridine (98.0%), acetic anhydride (98.0%), 5-(hydroxymethyl)furfural (HMF, 98%), 2,5-bis(hydroxymethyl)furan (BHMF, 95%), 5-methylfurfural (5-MF, 98%), 2,5-dimethylfuran (DMF, 99%), 2-(hydroxymethyl)-5-methylfuran (MFA, 95%), 2,5-dimethyltetrahydrofuran (DMTHF, 96%), were obtained from Sigma-Aldrich. Tetrahydrofuran (THF, 99%) and acetonitrile (99.99%) were supplied by Thermo Fisher.

Mesoporous carbon (CMC) was prepared from fructose (99% ADM) using $ZnCl_2$ as template and catalyst.[1] N-containing mesoporous carbon (NMC) was synthesized by carbonization of melamine (99%, Sigma-Aldrich) and formaldehyde (37 wt% in water, Sigma-Aldrich) using $CaCl_2$ (>95%, Fluka) as template.[2] As-prepared NMC and CMC were purified by 1.5 M HNO_3 aqueous solution at room temperature for 72 h followed by washing and filtration in deionized water several times until the pH of the filtrate became neutral. The purified NMC and CMC supports were dried at 80 °C overnight and were ground for further use. After purification, the amounts of the porogen residuals in the catalysts determined by ICP-MS are 0.04 wt% $CaCl_2$ for Pd/NMC and 0.06 wt% $ZnCl_2$ for Pd/CMC. Activated carbon (AC, Norit SX2) was obtained from Sigma-Aldrich as reference support.

Catalyst preparation

The supported Pd catalysts Pd/NMC, Pd/CMC, and Pd/AC were prepared using a sol-immobilization method. Briefly, an aqueous solution of $PdCl_2$ of the desired concentration was prepared. Subsequently, a polyvinyl alcohol (PVA) solution (1 wt%, MW = 9000 -10000 g/mol) was added (PVA/Pd (wt/wt) = 1.2). Then, a freshly prepared $NaBH_4$ aqueous solution (0.1 M) was added, which formed a dark-brown colloidal solution containing the Pd nanoparticles (NPs). After 30 min of sol generation, the powder support was added to the dark-brown colloidal solution at proper pH under vigorous stirring. The required amount of support was calculated to achieve a Pd loading of 1 wt%. After 2 h, the catalyst was obtained after filtration,

washing with distilled water, and drying at 80 °C overnight.

Characterization

XRD patterns were recorded using a Philips X'Pert MPD diffractometer with Cu Kα radiation and post-monochromator in a 2θ range from 5° to 80°. Elemental analysis of Pd was performed by AAS with a PerkinElmer AAS Model Analyst200 after acid digestion. ICP-MS measurements were performed with a iCAP RQ ASX-560 instrument to detect the leached Pd amount in the reaction solution. Scanning transmission electron microscopy (STEM) and transmission electron microscopy (TEM) measurements were carried out on a probe-side aberration-corrected JEOL JEM-2200FS with an acceleration voltage of 200kV. The effective area of the detector was 200 mm^2. The specimens for STEM and TEM were prepared by ultrasonically dispersing the powder samples in high-purity ethanol and then allowing a drop of the suspension to evaporate on a Au grid coated with carbon. Great care was taken to minimize the influence of electron beam damage on the derived Pd particle size distributions.

X-ray photoelectron spectroscopy (XPS) measurements were performed in an ultrahigh vacuum setup equipped with a high-resolution Gammadata Scienta SES 2002 analyzer. A monochromatic Al Kα X-ray source (1486.6 eV, anode operating at 14.5 kV and 30.5 mA) was used as incident radiation. The pressure inside the measuring chamber was kept in the range of 3.5 to 7×10^{-10} mbar during each measurement. The analyzer slit width was set at 0.3 mm and the pass energy was fixed at 200 eV for all measurements. The overall energy was better than 0.5 eV. Charging effects due to the insufficient conductivity of the carbon materials were mediated by using a flood gun (SPECS). All spectra were calibrated based on the C 1s binding energy of 284.5 eV. The CASA XPS program was used to analyze the XP spectra, and a mixed Gaussian-Lorentzian function and a Shirley background subtraction were applied in the fitting of the XPS data.

The TPR experiments were performed with a gas mixture of 2 % hydrogen in helium. The oven was cooled to 95 K with liquid nitrogen, before the feed was switched from pure helium to the reduction gas mixture. The oven was then heated with a constant heating ramp of 1 K/min to 673 K. Hydrogen consumption was recorded during heating to observe the reduction behavior

of the catalyst.

X-ray absorption spectroscopy (XAS) experiments were performed at PETRA III Extension beamline P65 (energy range: 4 - 44 keV) at DESY (Deutsches Elektronensynchrotron) in Hamburg (Germany).[3] For the measurements at the Pd K-edge, a Si(311) C-type double crystal monochromator was used. The beam current was 100 mA with a ring energy of 6.08 GeV. The samples were measured in glass capillaries without dilution. All spectra were recorded as continuous scans in fluorescence mode at ambient temperature and pressure in the range of -150 eV to 1000 eV around the edge within 180 sec. For calibration, a palladium foil was measured as a reference simultaneously with the samples.

The data treatment was performed using the Demeter software package.[4] In order to compensate for the oversampling of the continuous scan mode, the data points of the obtained spectra were reduced with the help of the 'rebin'-function of the Athena software (edge region: -50 to +50 eV; pre-edge grid: 5 eV; XANES grid: 0.5 eV; EXAFS grid: 0.05 Å-1). For data evaluation, a Victoreen-type polynomial was subtracted from the spectrum to remove the background using the Athena software. The first inflection point was taken as edge energy E0. No phase shift corrections have been applied. The EXAFS analysis was performed using the Artemis software. Prior to the fitting procedure, the amplitude reduction factor S02 and the Debye-Waller factor σ^2 (Pd-Pd) were determined for a Pd reference foil and used as a fixed parameter for all materials.

Catalytic tests

Hydrogenolysis of HMF with H_2. The catalytic performance of the catalysts for the hydrogenolysis of HMF was tested in a stainless-steel autoclave (Parr Autoclave 4560, 160 mL). Typically, 1.5 mmol HMF and 50 mg catalyst were added into the vessel precharged with 30 mL tetrahydrofuran. After purging with H_2, the reaction was performed at 160 °C, 5 bar initial pressure with a stirring speed of 600 rpm for 5 h. Liquid samples of 0.5 mL were taken via a sampling line after 1, 2, 3, 4, and 5 h. The liquid samples were filtered using membrane filters and then analyzed by gas chromatography (GC). GC analysis was performed using an Agilent 7820A GC system equipped with a DB-XLB column (30 nm × 0.18 nm × 0.18 μm) and

a FID detector. All analyses were performed three times each. Biphenyl was used as the internal standard and the carbon balance based on furan was in the range of 95 to 103 %. For easy comparison, quantification was achieved by using a normalization method. The errors of the measurements were calculated to be 3-4%.

Hydrogenolysis of HMF with formic acid. A similar procedure was applied to the conversion of HMF to DMF with formic acid as the hydrogen donor. Briefly, 1.5 mmol HMF, 50 mg catalyst, and 45 mmol formic acid (30 equiv.) were dissolved in 30 mL THF. After purging and pressurizing with 5 bar N_2, the reaction was conducted at 160 °C and 600 rpm. Liquid samples were taken periodically.

Hydrogenolysis of HMF with formic acid in the presence of H_2. A similar procedure was applied to the conversion of HMF to DMF with formic acid in the presence of external H_2. Briefly, 1.5 mmol HMF, 50 mg catalyst, and 45 mmol formic acid (30 equiv.) were dissolved in 30 mL THF. After purging and pressurizing with 5 bar H_2, the reaction was conducted at 160 °C and 600 rpm. Liquid samples were taken periodically.

Synthesis of 5-[(formyloxy)methyl]furfural (FMF) from HMF. In a 100-mL Schlenk flask, 16 mmol acetic anhydride and 16 mmol formic acid were stirred at 0 °C under Ar atmosphere for 1 h. A solution of 4 mmol HMF in 20 mL acetonitrile was added to the resulting solution via a syringe. After 20 min stirring, 0.8 mmol pyridine was added. The mixture was stirred for 1 h at 0 °C and additionally for 2 h at room temperature. Solvent and excess reagents were removed under vacuum to yield FMF as orange oil. ^{1}H NMR spectrum of FMF was recorded on Bruker AVIII-300 using $CDCl_3$ as the solvent. The spectrum shown in Figure S15 confirms the successful synthesis of FMF.

Hydrogenolysis of FMF to DMF with formic acid. In a typical experiment, 1.5 mmol FMF, 50 mg Pd/NMC, and 45 mmol formic acid (30 equiv.) were dissolved in 30 mL THF. After purging and pressurizing with 5 bar N_2, the reaction was conducted at 160 °C and 600 rpm. Liquid samples were taken periodically.

Reusability study

For catalytic reusability tests, the hydrogenolysis of HMF to DMF was performed with formic

acid in the presence of H_2 over Pd/NMC. After 3 h reaction, the catalyst was recycled from the reaction mixture by centrifugation, washing with THF and acetonitrile, and drying overnight at 80 °C. The recovered Pd/NMC catalyst was subsequently reused for the hydrogenolysis of HMF.

***In situ* ATR-IR spectroscopy**

In situ ATR-IR spectroscopy was employed to monitor the reaction progress and to investigate the reaction pathways. The hydrogenolysis of HMF to DMF over Pd/NMC was carried out in a 300 mL stainless-steel autoclave (Berghof BR-300), and the *in situ* ATR-IR spectra were recorded every 2 min using a Mettler Toledo ReactIR™ 15 spectrometer equipped with a 6.35 mm diameter Dicomp probe. Each spectrum was collected with a resolution of 4 cm^{-1} and 256 scans in the range of 650 to 4000 cm^{-1}.

In a typical run, 45 mmol HMF, 500 mg Pd/NMC catalyst, and the required amount of FA were dissolved in 120 mL THF. After purging with H_2, the reactor was pressurized with 10 bar H_2. The reaction was conducted at 160 °C and was monitored by *in situ* ATR-IR for 6 h. The reference spectra of the standard compounds (i.e., HMF, BHMF, 5-MF, and DMF) were recorded prior to the reaction separately.

Results

Characterization

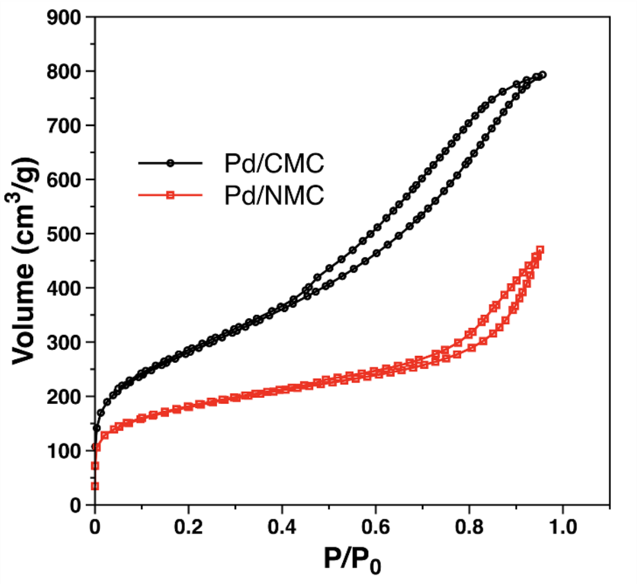

Figure S1. N_2 physisorption isotherms of Pd/NMC and Pd/CMC.

Table S1. Physicochemical properties of the carbon materials and supported catalysts.

Sample	Pd[a] [wt%]	S_{BET}[b] [m^2g^{-1}]	V_{pore} [cm^3g^{-1}]	D_{pore}[c] [nm]	$D_{particle}$[d] [nm]
NMC	–	657	0.7	4.9	–
CMC	–	997	1.2	5.0	–
AC	–	617	0.5	3.3	–
Pd/NMC	0.95	625	0.7	4.7	3.4
Pd/CMC	0.98	971	1.2	4.9	4.2
Pd/AC	0.98	473	0.4	3.4	7.8

[a] Pd loading determined by AAS; [b] Specific surface area (BET method); [c] Average pore diameter (BJH method); [d] Mean particle size from TEM.

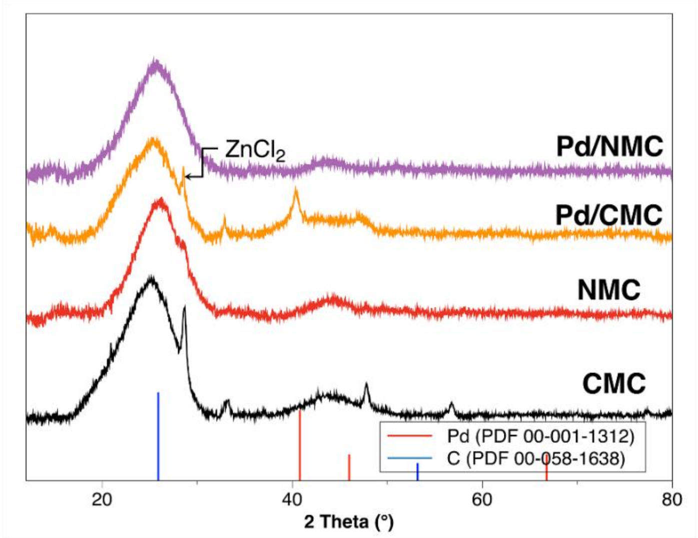

Figure S2. XRD patterns of Pd/NMC, Pd/CMC and the mesoporous carbon supports

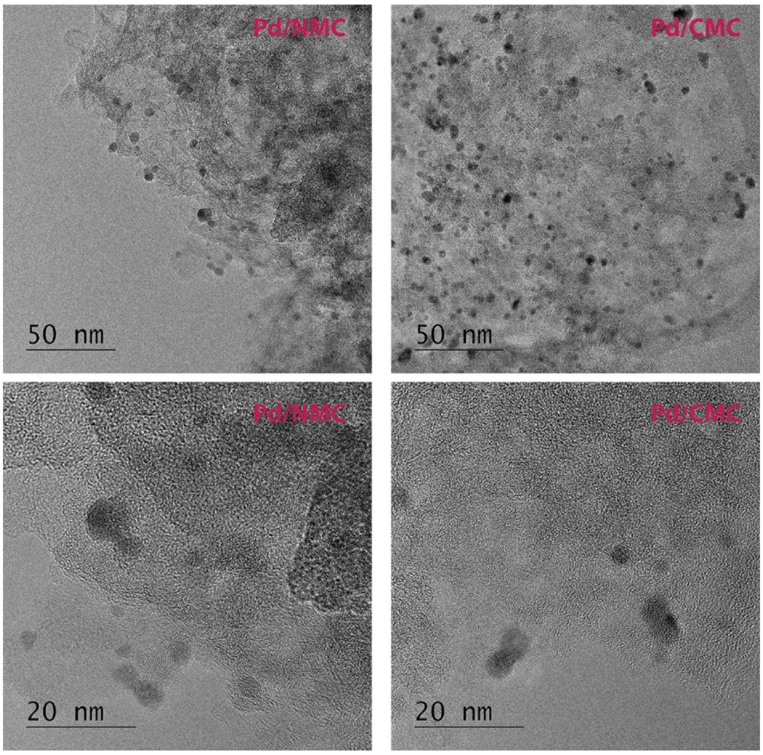

Figure S3. TEM images for Pd/CMC and Pd/NMC as prepared.

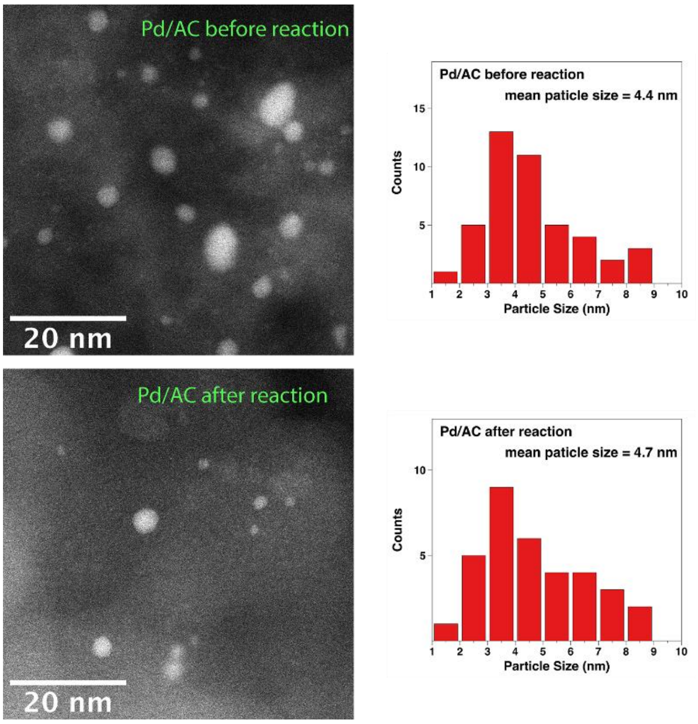

Figure S4. HAADF-STEM images and Pd particle size distributions for Pd/AC before and after reaction.

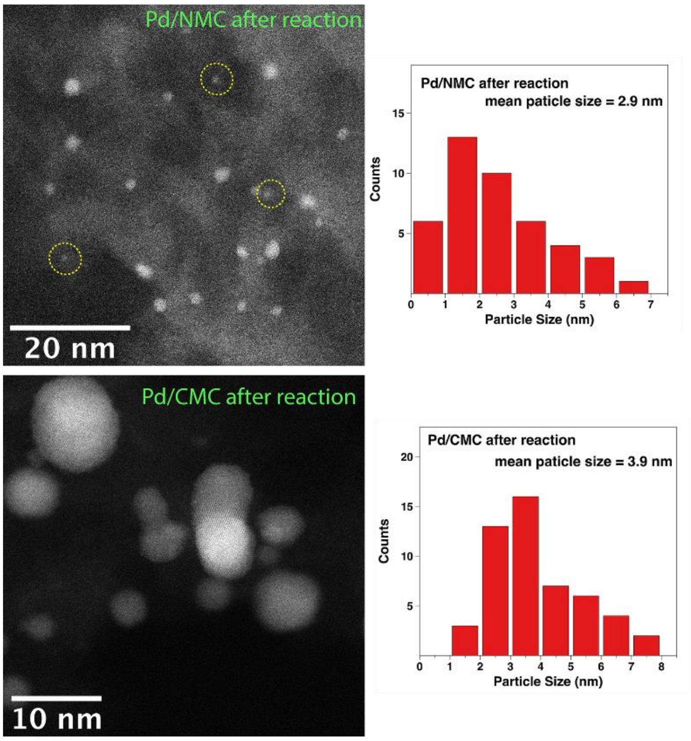

Figure S5. HAADF-STEM images and Pd particle size distributions for Pd/NMC and Pd/CMC after reaction in the presence of FA and H_2. The yellow circles refer to sub-1 nm Pd NPs or clusters.

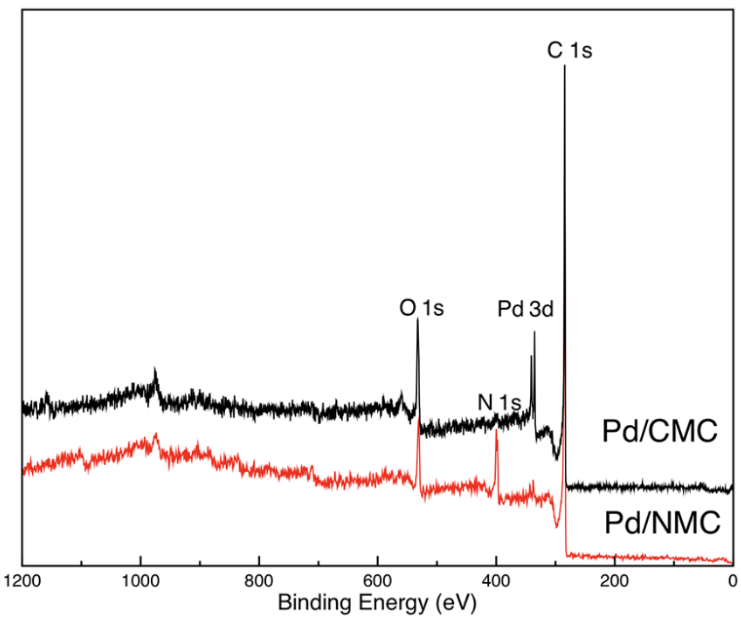

Figure S6. XP survey spectra of Pd/NMC and Pd/CMC as prepared.

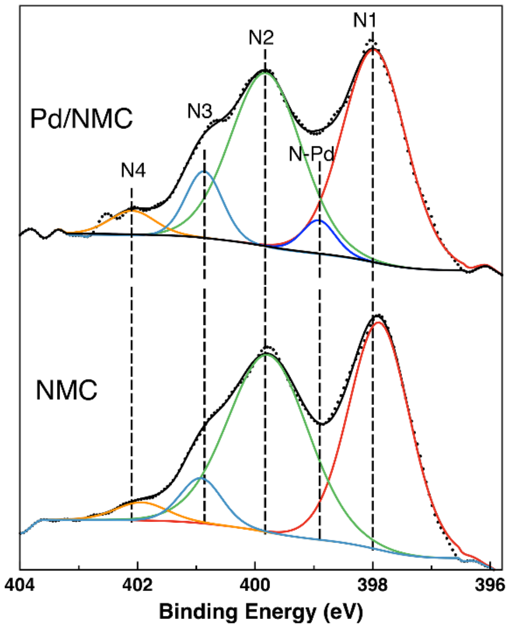

Figure S7. N 1s regions of the deconvoluted XPS results for NMC and Pd/NMC as prepared.[5]

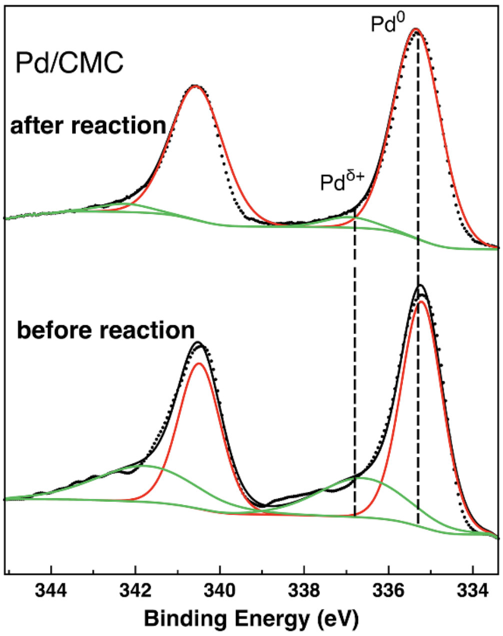

Figure S8. Pd 3d regions of the deconvoluted XPS results for Pd/CMC before and after reaction in the presence of FA and H_2. The XPS spectrum of Pd/CMC before reaction was reported in our previous report.[5]

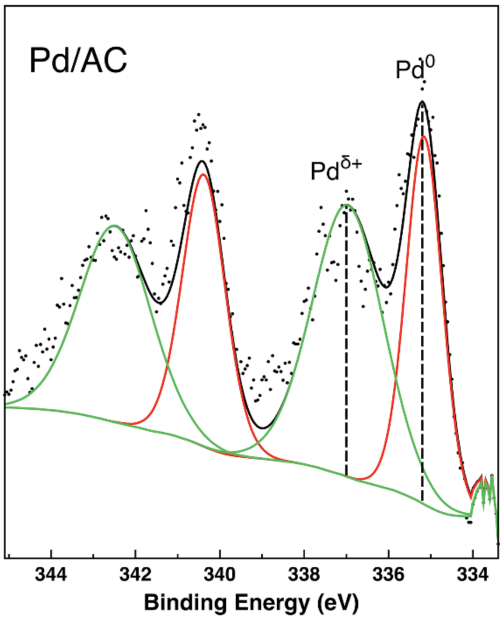

Figure S9. Pd 3d regions of the deconvoluted XPS results for Pd/AC as prepared.

Table S2. Relative abundance (%) of the components in the Pd $3d_{5/2}$ spectra

Sample	component/binding energy (eV)		
	Pd^0 335.0-335.5	$Pd^{\delta+}$ 336.5	Pd^{2+} 337.9
Pd/NMC before reaction	36	14	50
Pd/NMC after reaction	59	11	30
Pd/CMC before reaction*	68	32	–
Pd/CMC after reaction	91	9	–
Pd/AC before reaction	43	57	–

* The results of Pd/CMC before reaction was reported in our previous report[5]

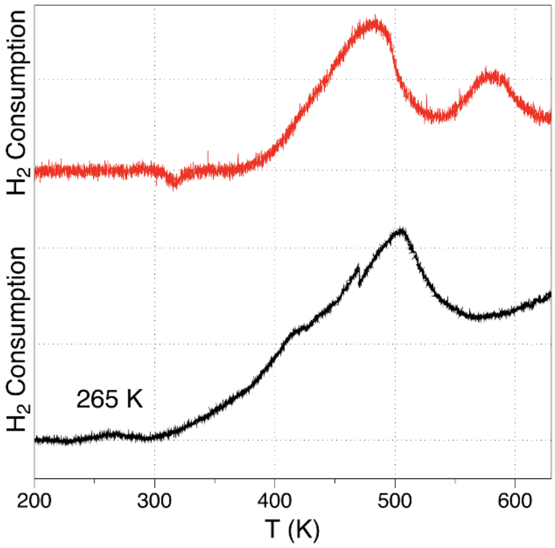

Figure S10. H_2 TPR profiles of as-prepared Pd/NMC (black line) and Pd/CMC (red line).

The H_2 TPR profiles of the Pd/NMC and Pd/CMC are shown in Figure S10. For Pd/NMC, a reduction peak at 265 K is observed, owing to the reduction of $Pd^{\delta+}$ and Pd^{2+} to Pd^0. By comparison, this reduction peak is not observed for Pd/CMC, probably because the Pd NPs in Pd/CMC are mostly in the reduced metallic state (see Table S1) and the Pd loading is low. The large and broad peaks with onset temperature of 300 K for Pd/NMC and 375 K for Pd/CMC are the reduction of the surface functional groups of NMC and CMC, respectively. There is one negative peak at 315 K, which originates from the decomposition of palladium hydride. This palladium hydride peak is not observed for Pd/NMC, probably due to the overlapping with the broad reduction peak of the surface functional groups.

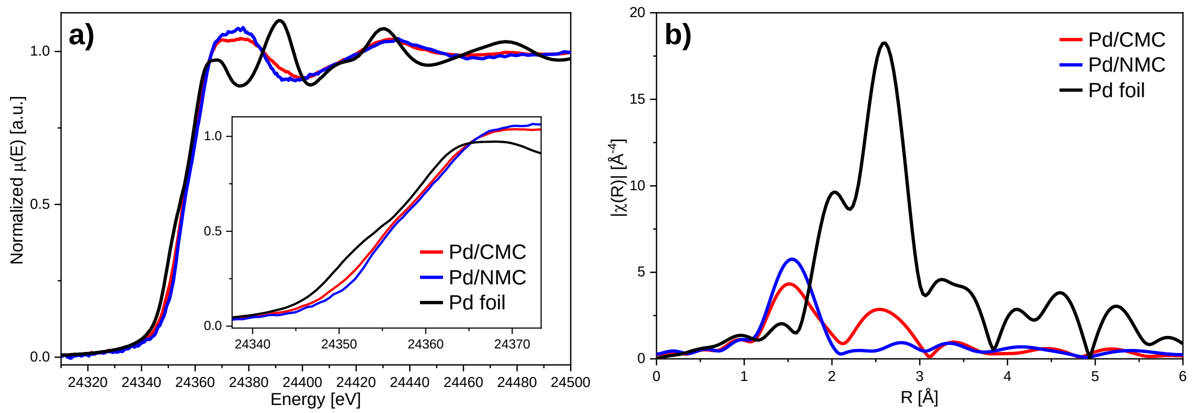

Figure S11. XANES (a) and Fourier-transformed EXAFS (b) spectra of as-prepared Pd/CMC and Pd/NMC in comparison to a Pd foil. Note that no phase shift corrections have been applied and the real distances are expected to be ≈ 0.4 Å larger.

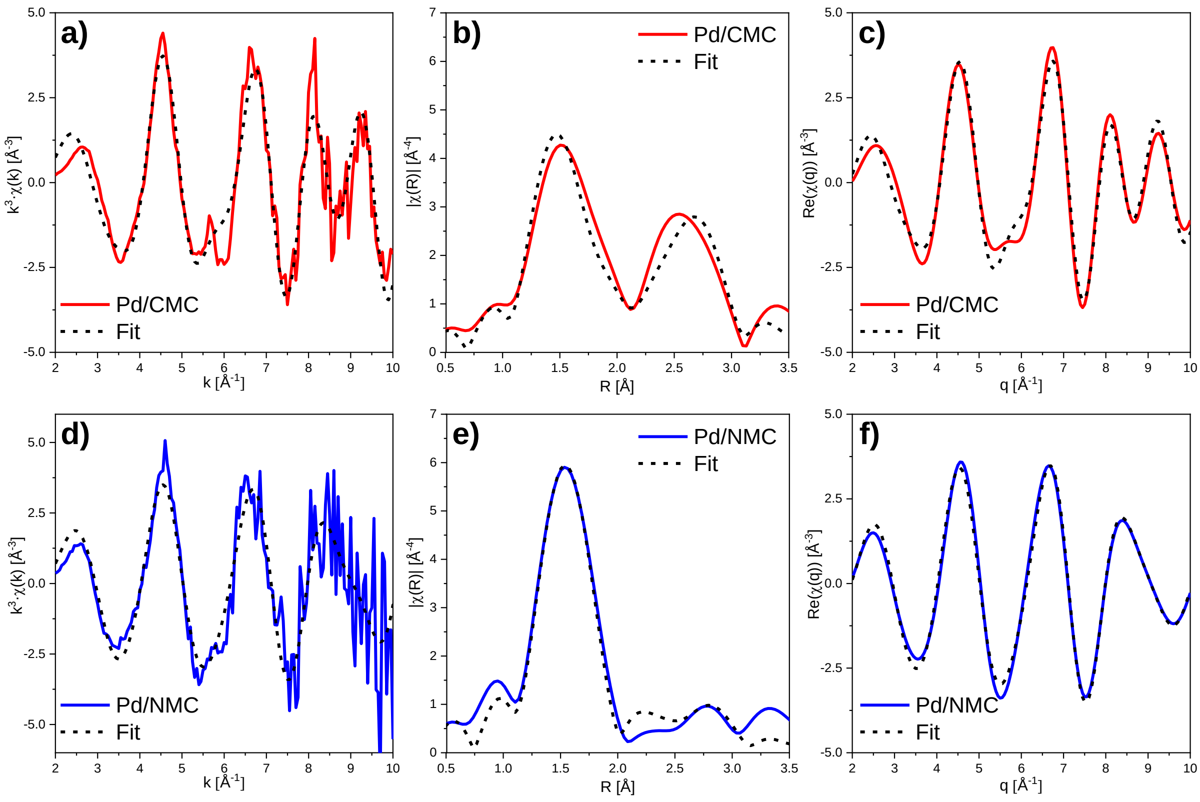

Figure S12. Comparison of experimentally obtained spectra and the corresponding best fit calculations of as-prepared Pd/CMC (**a** - **c**) and Pd/NMC (**d** - **f**) materials. EXAFS spectra (**a**, **d**), Fourier-transformed EXAFS spectra (**b**, **e**) and the backtransformation thereof (**c**, **f**).

Table S3. Best fit values of as-prepared Pd/CMC and Pd/NMC obtained by EXAFS structure fitting.

Sample	Abs-Bs[a]	N(Bs)[b]	R(Abs-Bs)[c] [Å]	σ^2 [d] [Å^2]
**Pd/CMC**	Pd – O	3.45 ± 0.85	2.02 ± 0.02	0.0062 ± 0.0034
	Pd – Pd	1.96 ± 0.31	2.76 ± 0.03	0.0048*
	S_0^2 [e]= 0.820; ΔE_0 [f] = 6.56 ± 2.56 eV; χ^2_{red}[g] = 17; R[h] = 0.034; N(path)[i] = 2; N(par)[j] = 6; k-range: 2 - 10; R-range: 1 - 3.			
**Pd/NMC**	Pd – N	4.38 ± 0.52	2.06 ± 0.02	0.0031 ± 0.0012
	Pd – Pd	0.80 ± 0.20	2.76 ± 0.03	0.0048*
	S_0^2 [e]= 0.820; ΔE_0 [f] = 7.97 ± 1.34 eV; χ^2_{red}[g] = 1; R[h] = 0.011; N(path)[i] = 2; N(par)[j] = 6; k-range: 2 - 10; R-range: 1 - 3.			

[a] Abs = X-ray absorbing atom, Bs = backscattering atom. [b] Number of backscattering atoms. [c] Distance between absorbing and backscattering atom. [d] Debye-Waller factor. [e] Amplitude reduction factor. [f] Accounts for the shift of E_0 between theory and experiment. [g] Reduced χ^2 error (considers the number of independent points and number of varied parameters besides the error to the experiment). [h] Fit index. [i] Total number of fitted paths including single and multiple scattering paths. [j] Number of free parameters used for the fit. * Used as fixed parameter

XAS spectra were recorded to confirm the oxidation state and to investigate the local chemical environment around the Pd centers. The XANES spectra of both samples were significantly different from metallic palladium and showed a significant whiteline indicating that a major amount of palladium atoms was present in an oxidic state (Figure S11**a**). Furthermore, the edge position shifted to higher energies by approximately 2.5 eV (inset Figure S11**a**), which also suggested the presence of oxidic Pd centers. However, a larger shift of the edge (≈ 5 eV) would be expected for a completely oxidized material containing only Pd^{2+}.[6] Therefore, both materials contain a mixture of Pd^{2+}/$Pd^{\delta+}$ and Pd^0. In accordance with the XPS results, the slightly higher intensity of the whiteline and the slightly larger shift of the edge indicate that a larger fraction of Pd is oxidized in Pd/NMC compared with Pd/CMC.

For Pd/CMC, the Fourier-transformed EXAFS spectra (Figure S11**b**) showed the presence of two shells of backscatterers at 1.54 Å and close to 2.6 Å (note that no phase shift corrections have been applied and the real distances are expected to be ≈ 0.4 Å larger). The comparison to the Pd foil suggested that the shell at 2.6 Å corresponds to a shell of Pd backscatterers in the metallic state. In contrast, the shell at 1.54 Å is expected to be formed by light backscatterers like carbon or oxygen, which would be expected for Pd species that are anchored to the support. For Pd/NMC, also a shell at 1.54 Å from presumably light backscatterers was clearly present.

However, the presence of a shell resulting from metallic Pd backscatterers cannot clearly be confirmed solely by comparison of the spectra. A comparison to a Fourier-transformed EXAFS spectrum of PdO[7] confirmed that no PdO clusters were present in either sample.

In addition to the qualitative evaluation of the Fourier-transformed EXAFS spectra, we performed an EXAFS structure fitting to derive quantitative information about the local chemical environment around the Pd centers. For both materials, the best fits were obtained assuming two different shells of backscatterers (Table S3). The first shell consisted of light backscatterers (N, O or C) and was found at 2.02 and 2.06 Å for Pd/CMC and Pd/NMC, respectively. Although we have assumed oxygen and nitrogen neighbors for the respective materials during the fitting procedure, this technique cannot distinguish these elements due to their similar backscattering properties. The numbers of backscatterers close to 4 is in accordance with a square-planar coordination geometry, which is typically found for many palladium complexes. The second shell was found at 2.76 Å for both materials, which is consistent with Pd backscatterers in a Pd^0 crystal lattice. The number of Pd backscatterers (1.96 for Pd/CMC, 0.80 for Pd/NMC) is small compared to a bulk Pd crystal (expected: 12.0). Note that the small number of Pd backscatterers for Pd/NMC is significant to obtain a good fit. As the number of backscatterers is only an average number and strongly depends on the cluster diameter,[8] the rather small number of Pd backscatterers suggests that very small Pd clusters or even isolated single atoms, which have a coordination number of zero, may be present. The smaller number of Pd backscatterers for Pd/NMC in comparison to Pd/CMC indicates a larger fraction of Pd clusters or isolated single atoms in Pd/NMC than in Pd/CMC, which is in good agreement with the STEM measurements and explains the differences in the Pd reflections in their XRD patterns. Furthermore, a size approximation based on the EXAFS structure fitting was not reasonable, since it cannot fully be excluded that some isolated Pd^{2+} single atom species are present in the material, which would adversely affect the size determination. Nonetheless, the small number of backscatterers and the low intensity of the shell at 2.6 Å in the Fourier-transformed EXAFS spectra support the assumption of small Pd clusters in the lower nanometer regime.

Catalytic results

Table S4. Catalytic activity of different catalysts on HMF hydrogenolysis to DMF

Entry	Catalysts	Reactant	Hydrogen Source	Time (h)	T (°C)	Solvent	Conversion (mol %)	S_{DMF} (mol %)	TOF (h^{-1})	Ref.
1	Pd/NMC	HMF	FA+H_2	2	160	THF	> 99.9	> 97.0	150	T[a]
2	Pd/NMC	HMF	FA	3	160	THF	60.8	64.3	41	T[a]
3	Pd/CMC	HMF	FA+H_2	3	160	THF	90.1	80.0	75	T[a]
4	Pd/CMC	HMF	FA	3	160	THF	19.4	62.9	13	T[a]
5	Pd/C	FMF	FA	15	120	dioxane	> 95.0	> 95.0	1.2×10^{-3}	9
6	Pd/C	FMF	FA+H_2	15	120	dioxane	> 95.0	87.6	0.27×10^{-3}	9
7	Pd/C/Zn	HMF	H_2	8	150	THF	> 99.9	85.0	27.2	10
8	Ni-Co/C	HMF	FA	24	210	THF	> 99.0	90.0	0.1	11
9	Ru/MoO_x/C	HMF	H_2	1	180	n-butyl alcohol	> 99.0	79.8	21.3	12

[a] This work, reaction conditions: 1.5 mmol HMF, 50 mg catalysts, 30 mL THF, 160 °C, 2 or 3 h;

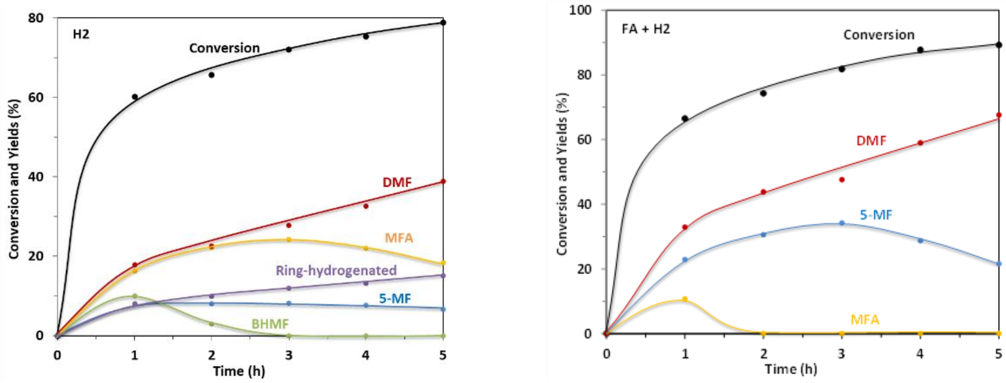

Figure S13. Time course of the hydrogenolysis of HMF to DMF over Pd/CMC with H_2 and with FA + H_2.

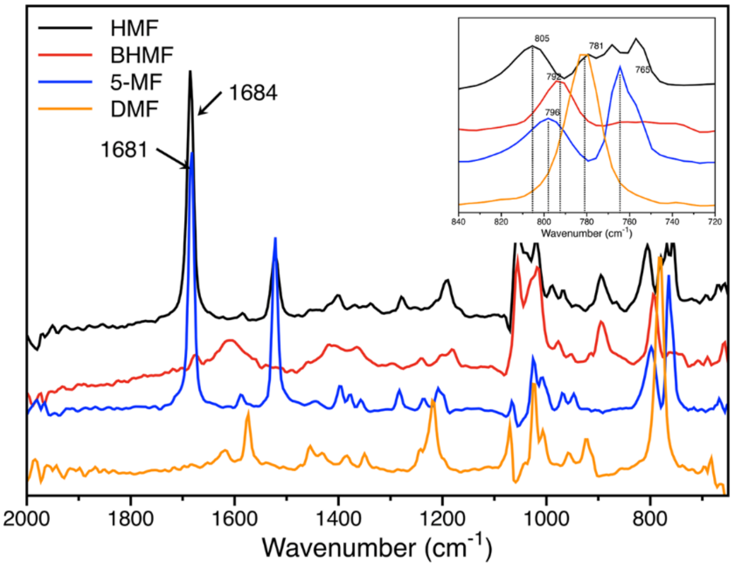

Figure S14. ATR-IR spectra of standard samples at 0.1 M in THF

Table S5. ATR-IR absorption bands of the standard samples in THF

	**ν(C=O) (cm^{-1})**	**ν(C=C) (cm^{-1})**	**ν(C−H) (cm^{-1})**
**HMF**	**1684**	**1520**	**805,779,767,756**
**BHMF**	**-**	**1608**	**792**
**5-MF**	**1681**	**1520**	**796,765**
**DMF**	**-**	**1573**	**781**

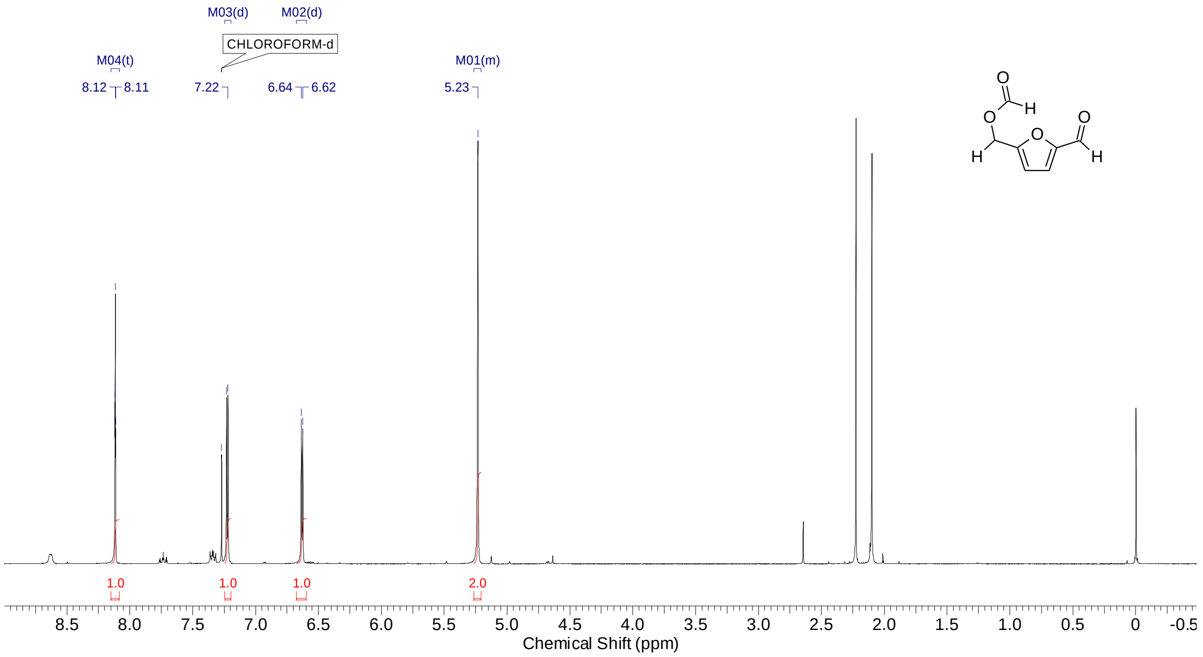

Figure S15. ^{1}H NMR spectrum of FMF, ^{1}H NMR (300 MHz, *CHLOROFORM-d*) δ ppm 8.65 (s, 1 H), 8.12 (t, *J*=0.9 Hz, 1 H), 7.22 (d, *J*=3.6 Hz, 1 H), 6.63 (d, *J*=3.5 Hz, 1 H), 5.21 - 5.26 (m, 2 H).

Computational

Methodology

The quantum chemical study was performed using density functional theory (DFT) with the TPSS[13] functional and def2-SVP[14] basis set, which includes Stuttgart-Cologne effective core potentials (def2-ecp)[15] for the Pd atoms. All calculations were carried out using the TURBOMOLE[16] program package with the multipole accelerated resolution of identity (MARI-J)[17] approximation with optimized auxiliary basis sets[18] and Grimme's D3[19] correction for London dispersion interactions.

The model of Pd/CMC, a Pd_{21} cluster on a hydrogen terminated graphene layer of 150 carbon atoms, has been shown to be a reasonable choice in a previous study.[20] For the bifunctional Pd/NMC system, three carbon atoms of the Pd/CMC system were replaced by nitrogen atoms. The most stable structures of these models as well as the minima of formic acid and hydrogen on these systems were determined by ground-state geometry optimizations. For relative energies, the zero-point vibrational energy (ZPVE) corrections from vibrational analysis were included. As the potential energy surfaces of the Pd/CMC and Pd/NMC systems are often very flat, the vibrational analysis of some systems still shows one (very small) negative frequency. We expect the errors introduced by this to be negligible. For determining reaction pathways and locating initial transition state structures the chain-of-state method implemented in the woelfling module of TURBOMOLE[21] was used. The initial transition state structures were then further optimized with the eigenvalue following trust-region image minimization (TRIM)[22] algorithm and validated by intrinsic reaction coordinate (IRC)[23] calculations. Adsorption energies and dissociation energies are provided with a negative sign, while energy barriers like activation barriers have a positive sign. Atomic charges were determined using natural population analyses (NPAs).[24]

Table S6. Adsorption energies of FA on Pd_{21}

Model system	FA configuration	ΔE_{ads} / kJ mol^{-1}
Pd_{21}/CMC	C=O binding	-129.6
Pd_{21}/CMC	C atom binding	-75.5
Pd_{21}/NMC	C=O binding	-132.5
Pd_{21}/NMC	C atom binding	not stable[a]

[a] For the C atom binding on Pd_{21}/NMC only configurations with imaginary frequencies were found.

Table S7. Dissociation energies of H_2 on Pd_{21} in kJ mol^{-1}

Model system	H atom configuration	TPSS/ def2-SVP	TPSS/ def2-TZVP	PBE/ def2-SVP
Pd_{21}/CMC	2 H in fcc positions	-111.1	-94.9	-122.0
Pd_{21}/CMC	1 H in fcc, 1 H at the edge	-96.0	-86.0	-116.8
Pd_{21}/NMC	2 H in fcc positions	-118.9	-112.4	-131.2
Pd_{21}/NMC	1 H in fcc, 1 H at the edge (close to Pd^{2+})	-116.9	-111.2	-129.1
Pd_{21}/NMC	1 H in fcc, 1 H at the edge (far from Pd^{2+})	-112.6	-108.2	-126.1

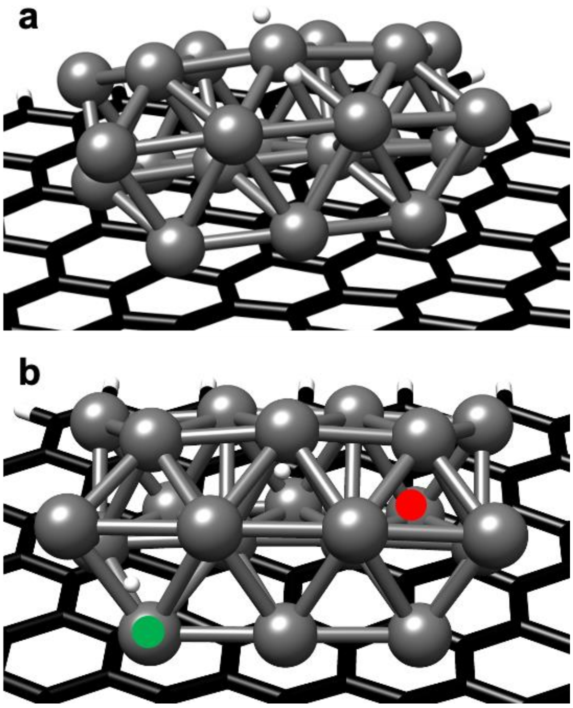

Figure S16. H atom configurations on Pd_{21}/CMC: (a) 2 H in fcc positions; (b) 1 H in fcc, 1 H at the edge. For Pd_{21}/NMC: lower left Pd atom (green; close) or back right Pd atom (red; far) is connected to 2 pyridinic N atoms.

References

1. Y. A. Huang, S. Hu, S. Zuo, Z. Xu, C. Han, J. Shen, *J. Mater. Chem.* **2009**, *19*, 7759-7764.
2. Y. A. Huang, F. Yang, Z. Xu, J. Shen, *J. Colloid Interf. Sci* **2011**, *363*, 193-198.
3. E. Welter, R. Chernikov, M. Herrmann, R. Nemausat, *AIP Conf. Proc.* **2019**, *2054*, 04002.
4. B. Ravel, M. Newville, *J. Synchrotron Rad.* **2005**, *12*, 537-541
5. L. Warczinski, B. Hu, T. Eckhard, B. Peng, M. Muhler, C. Hättig, *Phys. Chem. Chem. Phys.* **2020**, *https://doi.org/10.1039/D0CP03234D*.
6. K. A. Karinshak, P. Lott, M. P. Harold, O. Deutschmann, *ChemCatChem* **2020**, *12*, 3712-3720.
7. K. Okumura, J. Amano, N. Yasunobu, M. Niwa, *J. Phys. Chem. B* **2000**, *104*, 1050–1057.
8. A. Jentys, *Phys. Chem. Chem. Phys.* **1999**, *1*, 4059-4063
9. J. Mitra, X. Zhou, T. Rauchfuss, *Green Chem.* **2015,** *17*, 307-313.
10. B. Saha, C. M. Bohn, M. M. Abu-Omar, *ChemSusChem* **2014,** *7*, 3095-3101.
11. P. Yang, Q. Xia, X. Liu, Y. Wang, *Fuel* **2017,** *187*, 159-166.
12. Y. Yang, Q. Liu, D. Li, J. Tan, Q. Zhang, C. Wang, L. Ma, *RSC Advances* **2017,** *7*, 16311-16318.
13. J. Tao, J. P. Perdew, V. N. Staroverov, G. E. Scuseria, *Phys. Rev. Lett.* **2003,** *91* (14), 146401.
14. F. Weigend, R. Ahlrichs, *Phys. Chem. Chem. Phys.* **2005,** *7* (18), 3297-3305.
15. D. Andrae, U. Häußermann, M. Dolg, H. Stoll, H. Preuß, *Theor. Chim. Acta* **1990,** *77* (2), 123-141.
16. Turbomole V7.3 2018, a development of University of Karlsruhe and Forschungszentrum Karlsruhe GmbH, 1989-2007, TURBOMOLE GmbH, since 2007, available from http://www.turbomole.com.
17. M. Sierka, A. Hogekamp, R. Ahlrichs, R., *J. Chem. Phys.* **2003,** *118* (20), 9136-9148.
18. F. Weigend, *Phys. Chem. Chem. Phys.* **2006,** *8* (9), 1057-1065.
19. S. Grimme, J. Antony, S. Ehrlich, H. Krieg, *J. Chem. Phys.* **2010,** *132* (15), 154104.
20. L. Warczinski, C. Hättig, *Phys. Chem. Chem. Phys.* **2019,** *21* (38), 21577-21587.
21. P. Plessow, *J. Chem. Theory Comput.* **2013,** *9* (3), 1305-1310.
22. T. Helgaker, *Chem. Phys. Lett.* **1991,** *182* (5), 503-510.
23. K. Fukui, *Acc. Chem. Res.* **1981,** *14* (12), 363-368.
24. A. E. Reed, R. B. Weinstock, F. Weinhold, *J. Chem. Phys.* **1985,** *83* (2), 735-746.

Liste der Abkürzungen

HMF	5-(Hydroxymethyl)furfural
LA	Levulinsäure
FDCA	2,5-Furan-Dicarbonsäure
EL	Ethyl-Levulinat
DMF	2,5-Dimethylfuran
DFF	2,5-Diformylfuran
FA	Ameisensäure
BHMF	2,5-Bis(hydroxymethyl)furan
5-MF	5-Methylfurfural
MFA	2-(Hydroxymethyl)-5-methylfuran
DMTHF	2,5-Dimethyltetrahydrofuran
FMF	5-[(Formyloxy)methyl]furfural
BHMTHF	2,5-Bis(hydroxymethyl)tetrahydrofuran
MTHFA	5-Methyltetrahydrofurfurylalkohol

Experimentelles

Materialien

Alle kommerziell erhältlichen Reagenzien wurden wie erhalten ohne weitere Reinigung verwendet, sofern nicht anders angegeben. Palladiumchlorid (99 %), Ameisensäure (97 %), Eisessigsäure, Pyridin (98,0 %), Essigsäureanhydrid (98,0 %), 5-(Hydroxymethyl)furfural (HMF, 98 %), 2,5-Bis(hydroxymethyl)furan (BHMF, 95%), 5-Methylfurfural (5-MF, 98 %), 2,5-Dimethylfuran (DMF, 99 %), 2-(Hydroxymethyl)-5-methylfuran (MFA, 95 %), 2,5-Dimethyltetrahydrofuran (DMTHF, 96%), wurden von Sigma-Aldrich bezogen. Tetrahydrofuran (THF, 99 %) und Acetonitril (99,99 %) wurden von Thermo Fisher geliefert. Mesoporöser Kohlenstoff (CMC) wurde aus Fruktose (99 % ADM) unter Verwendung von $ZnCl_2$ als Vorlage und Katalysator hergestellt.[1] N-haltiger mesoporöser Kohlenstoff (NMC) wurde durch Karbonisierung von Melamin (99 %, Sigma-Aldrich) und Formaldehyd (37 Gew.-% in Wasser, Sigma-Aldrich) unter Verwendung von CaCl2 (>95 %, Fluka) als Vorlage synthetisiert.[2] Die so hergestellten NMC und CMC wurden mit 1,5 M HNO_3 wässriger Lösung bei Raumtemperatur für 72 h gereinigt, gefolgt von mehrmaligem Waschen und Filtration in deionisiertem Wasser, bis der pH-Wert des Filtrats neutral wurde. Die gereinigten NMC- und CMC-Träger wurden über Nacht bei 80 °C getrocknet und für die weitere Verwendung gemahlen. Nach der Reinigung betragen die mittels ICP-MS bestimmten Mengen der Porogenrückstände in den Katalysatoren 0,04 Gew.-% $CaCl_2$ für Pd/NMC und 0,06 Gew.-% $ZnCl_2$ für Pd/CMC. Aktivkohle (AC, Norit SX2) wurde von Sigma-Aldrich als Referenzträger bezogen.

Katalysator Präparation

Die geträgerten Pd-Katalysatoren Pd/NMC, Pd/CMC und Pd/AC wurden mit einer Sol-Immobilisierungsmethode hergestellt. Kurz zusammengefasst wurde eine wässrige Lösung von $PdCl_2$ in der gewünschten Konzentration hergestellt. Anschließend wurde eine Polyvinylalkohol (PVA)-Lösung (1 Gew.-%, MW = 9000 -10000 g/mol) hinzugefügt (PVA/Pd (Gew./Gew.) = 1,2). Dann wurde eine frisch hergestellte wässrige $NaBH_4$-Lösung (0,1 M) zugegeben, die eine dunkelbraune kolloidale Lösung bildete, die die Pd-Nanopartikel (NPs)

enthielt. Nach 30 Minuten Solbildung wurde der Pulverträger bei richtigem pH-Wert unter kräftigem Rühren zu der dunkelbraunen kolloidalen Lösung gegeben. Die erforderliche Menge des Trägers wurde berechnet, um eine Pd-Beladung von 1 Gew.-% zu erreichen. Nach 2 h wurde der Katalysator nach Filtration, Waschen mit destilliertem Wasser und Trocknen bei 80 °C über Nacht erhalten.

Charakterisierung

Pulverdiffraktogramme wurden mit einem Philips X'Pert MPD-Diffraktometer mit Cu Kα-Strahlung und Post-Monochromator in einem 2θ-Bereich von 5° bis 80° aufgenommen. Die Elementaranalyse von Pd wurde mittels AAS mit einem Perkin Elmer AAS Model Analyst 200 nach Säureaufschluss durchgeführt. ICP-MS-Messungen wurden mit einem iCAP RQ ASX-560 Gerät durchgeführt, um die ausgewaschene Pd-Menge in der Reaktionslösung zu bestimmen. Rastertransmissionselektronenmikroskopie- (STEM) und Transmissionselektronenmikroskopie- (TEM) Messungen wurden mit einem probenseitig aberrationskorrigierten JEOL JEM-2200FS mit einer Beschleunigungsspannung von 200 kV durchgeführt. Die effektive Fläche des Detektors betrug 200 mm^2. Die Proben für STEM und TEM wurden hergestellt, indem die Pulverproben mit Ultraschall in hochreinem Ethanol dispergiert und dann ein Tropfen der Suspension auf einem mit Kohlenstoff beschichteten Au-Gitter verdampft wurde. Es wurde darauf geachtet, dass der Einfluss von Elektronenstrahlschäden auf die bestimmten Pd-Partikelgrößenverteilungen minimiert wurde.

Röntgen-Photoelektronen-Spektroskopie (XPS)-Messungen wurden in einer Ultrahochvakuum-Anlage durchgeführt, die mit einem hochauflösenden Gammadata Scienta SES 2002-Analysator ausgestattet war. Als Anregungsstrahlung wurde eine monochromatische Al Kα-Röntgenquelle (1486,6 eV, Anode bei 14,5 kV und 30,5 mA) verwendet. Der Druck in der Messkammer war bei jeder Messung im Bereich von 3,5 bis 7×10^{-10} mbar. Die Spaltbreite des Analysators wurde auf 0,3 mm eingestellt und die Durchlassenergie wurde für alle Messungen auf 200 eV festgelegt. Die Energieauflösung war besser als 0,5 eV. Aufladungseffekte aufgrund der unzureichenden Leitfähigkeit der Kohlenstoffmaterialien wurden durch den Einsatz einer niederenergetischen Elektronenquelle (SPECS) kompensiert.

Alle Spektren wurden auf Basis der C 1s-Bindungsenergie von 284,5 eV kalibriert. Das CASA XPS-Programm wurde zur Analyse der XP-Spektren verwendet, und eine gemischte Gauß-Lorentz-Funktion und eine Shirley-Hintergrundsubtraktion wurden bei der Anpassung der XPS-Daten angewendet.

Die TPR-Experimente wurden mit einem Gasgemisch aus 2 % Wasserstoff in Helium durchgeführt. Der Ofen wurde mit flüssigem Stickstoff auf 95 K gekühlt, bevor die Zufuhr von reinem Helium auf das Reduktionsgasgemisch umgeschaltet wurde. Der Ofen wurde dann mit einer konstanten Heizrampe von 1 K/min auf 673 K aufgeheizt. Der Wasserstoffverbrauch wurde während des Heizens aufgezeichnet, um das Reduktionsverhalten des Katalysators zu beobachten.

Experimente zur Röntgenabsorptionsspektroskopie (XAS) wurden an der PETRA III Extension Beamline P65 (Energiebereich: 4 - 44 keV) am DESY (Deutsches Elektronensynchrotron) in Hamburg durchgeführt.[3] Für die Messungen an der Pd-K-Kante wurde ein Si(311) C-Typ-Doppelkristall-Monochromator verwendet. Der Strahlstrom betrug 100 mA bei einer Ringenergie von 6,08 GeV. Die Proben wurden in Glaskapillaren ohne Verdünnung gemessen. Alle Spektren wurden als kontinuierliche Scans im Fluoreszenzmodus bei Umgebungstemperatur und -druck im Bereich von -150 eV bis 1000 eV um den Rand herum innerhalb von 180 s aufgenommen. Zur Kalibrierung wurde gleichzeitig mit den Proben eine Palladiumfolie als Referenz gemessen.

Die Datenaufbereitung erfolgte mit dem Demeter-Softwarepaket.[4] Um das Oversampling des kontinuierlichen Scanmodus zu kompensieren, wurden die Datenpunkte der erhaltenen Spektren mit Hilfe der 'rebin'-Funktion der Athena-Software reduziert (Kantenbereich: -50 bis +50 eV; Pre-Kanten-Gitter: 5 eV; XANES-Gitter: 0,5 eV; EXAFS-Raster: 0.05 Å-1). Zur Datenauswertung wurde ein Victoreen-Typ-Polynom vom Spektrum subtrahiert, um den Hintergrund mit Hilfe der Athena-Software zu entfernen. Der erste Wendepunkt wurde als Kantenenergie E0 genommen. Es wurden keine Korrekturen der Phasenverschiebung angewendet. Die EXAFS-Analyse wurde mit der Artemis-Software durchgeführt. Vor der Anpassungsprozedur wurden der Amplitudenreduktionsfaktor S02 und der Debye-Waller-Faktor σ^2 (Pd-Pd) für eine Pd-Referenzfolie bestimmt und als fester Parameter

für alle Materialien verwendet.

Katalytische Tests

Hydrogenolyse von HMF mit H_2. Die katalytische Aktivität der Katalysatoren für die Hydrogenolyse von HMF wurde in einem Edelstahlautoklaven (Parr Autoclave 4560, 160 mL) getestet. Typischerweise wurden 1,5 mmol HMF und 50 mg Katalysator in das mit 30 mL Tetrahydrofuran vorgefüllte Gefäß gegeben. Nach dem Spülen mit H_2 wurde die Reaktion bei 160 ℃, 5 bar Anfangsdruck mit einer Rührgeschwindigkeit von 600 U/min für 5 h durchgeführt. Flüssigproben von 0,5 mL wurden über eine Probenentnahmeleitung nach 1, 2, 3, 4 und 5 h entnommen. Die Flüssigproben wurden mit Membranfiltern filtriert und dann mittels Gaschromatographie (GC) analysiert. Die GC-Analyse wurde mit einem Agilent 7820A GC-System durchgeführt, das mit einer DB-XLB-Säule (30 nm × 0,18 nm × 0,18 µm) und einem FID-Detektor ausgestattet war. Alle Analysen wurden jeweils dreimal durchgeführt. Biphenyl wurde als interner Standard verwendet und die Kohlenstoffbilanz auf Basis von Furan lag im Bereich von 95 bis 103 %. Für einen einfachen Vergleich wurde die Quantifizierung mit Hilfe einer Normalisierungsmethode durchgeführt. Die Fehler der Messungen wurden mit 3-4 % berechnet.

Hydrogenolyse von HMF mit Ameisensäure. Ein ähnliches Verfahren wurde für die Umwandlung von HMF zu DMF mit Ameisensäure als Wasserstoffquelle angewandt. Kurz zusammengefasst wurden 1,5 mmol HMF, 50 mg Katalysator und 45 mmol Ameisensäure (30 Äquiv.) in 30 mL THF gelöst. Nach Spülung und Druckerhöhung mit 5 bar N_2 wurde die Reaktion bei 160 ℃ und 600 U/min durchgeführt. Es wurden periodisch Flüssigkeitsproben entnommen.

Hydrogenolyse von HMF mit Ameisensäure in Gegenwart von H_2. Ein ähnliches Verfahren wurde für die Umwandlung von HMF zu DMF mit Ameisensäure in Gegenwart von externem H_2 angewendet. 1,5 mmol HMF, 50 mg Katalysator und 45 mmol Ameisensäure (30 Äquiv.) wurden in 30 mL THF gelöst. Nach Spülung und Druckerhöhung mit 5 bar H_2 wurde die Reaktion bei 160 ℃ und 600 U/min durchgeführt. Es wurden periodisch Flüssigkeitsproben

entnommen.

Synthese von 5-[(Formyloxy)methyl]furfural (FMF) aus HMF. In einem 100-mL-Schlenkkolben wurden 16 mmol Essigsäureanhydrid und 16 mmol Ameisensäure bei 0 °C unter Ar-Atmosphäre 1 h lang gerührt. Eine Lösung von 4 mmol HMF in 20 mL Acetonitril wurde der resultierenden Lösung über eine Spritze zugegeben. Nach 20 min Rühren wurden 0,8 mmol Pyridin zugegeben. Die Mischung wurde für 1 h bei 0 °C und zusätzlich für 2 h bei Raumtemperatur gerührt. Lösungsmittel und überschüssige Reagenzien wurden unter Vakuum entfernt, um FMF als orangefarbenes Öl zu erhalten. Das ^{1}H-NMR-Spektrum von FMF wurde auf einem Bruker AVIII-300 mit $CDCl_3$ als Lösungsmittel aufgenommen. Das in Abbildung S15 gezeigte Spektrum bestätigt die erfolgreiche Synthese von FMF.

Hydrogenolyse von FMF zu DMF mit Ameisensäure. In einem typischen Experiment wurden 1,5 mmol FMF, 50 mg Pd/NMC und 45 mmol Ameisensäure (30 Äquiv.) in 30 mL THF gelöst. Nach Spülung und Druckerhöhung mit 5 bar N_2 wurde die Reaktion bei 160 ℃ und 600 U/min durchgeführt. Es wurden periodisch Flüssigkeitsproben entnommen.

Studie zur Wiederverwendbarkeit

Für katalytische Wiederverwendungstests wurde die Hydrogenolyse von HMF zu DMF mit Ameisensäure in Gegenwart von H_2 über Pd/NMC durchgeführt. Nach 3 h Reaktion wurde der Katalysator durch Zentrifugation, Waschen mit THF und Acetonitril und Trocknen über Nacht bei 80 ℃ aus dem Reaktionsgemisch zurückgewonnen. Der zurückgewonnene Pd/NMC-Katalysator wurde anschließend für die Hydrogenolyse von HMF wiederverwendet.

***In situ*-ATR-IR-Spektroskopie**

Die *In-situ*-ATR-IR-Spektroskopie wurde zur Überwachung des Reaktionsfortschritts und zur Untersuchung der Reaktionswege eingesetzt. Die Hydrogenolyse von HMF zu DMF über Pd/NMC wurde in einem 300-mL-Edelstahl-Autoklaven (Berghof BR-300) durchgeführt und die ATR-IR-Spektren wurden *in situ* alle 2 min mit einem Mettler Toledo ReactIR™ 15-Spektrometer aufgenommen, das mit einer Dicomp-Sonde mit 6,35 mm Durchmesser

ausgestattet war. Jedes Spektrum wurde mit einer Auflösung von 4 cm^{-1} und 256 Scans im Bereich von 650 bis 4000 cm^{-1} aufgenommen.

In einem typischen Experiment wurden 45 mmol HMF, 500 mg Pd/NMC-Katalysator und die erforderliche Menge FA in 120 mL THF gelöst. Nach dem Spülen mit H_2 wurde der Reaktor mit 10 bar H_2 unter Druck gesetzt. Die Reaktion wurde bei 160 °C durchgeführt und mittels *In-situ*-ATR-IR für 6 h verfolgt. Die Referenzspektren der Standardverbindungen (d. h. HMF, BHMF, 5-MF und DMF) wurden vor der Reaktion separat aufgenommen.

Ergebnisse

Charakterisierung

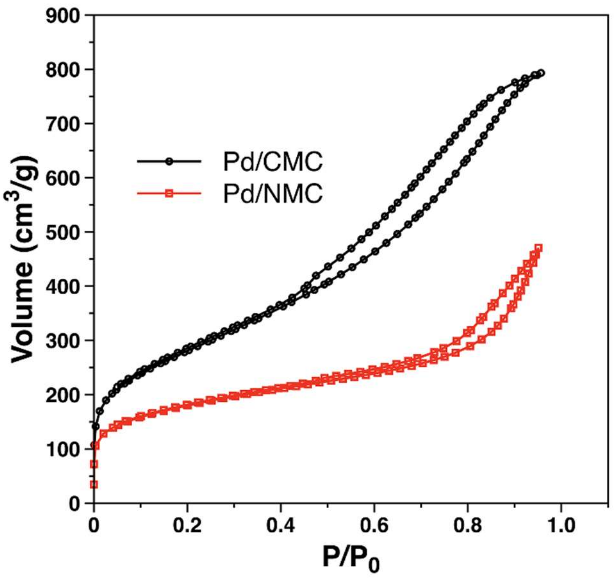

Abbildung S1. N_2-Physisorptionsisothermen von Pd/NMC und Pd/CMC.

Tabelle S1. Physikalisch-chemische Eigenschaften der Kohlenstoffmaterialien und Trägerkatalysatoren.

Probe	Pd[a] [wt%]	S_{BET}[b] [m^2g^{-1}]	V_{Pore} [cm^3g^{-1}]	D_{Pore}[c] [nm]	$D_{Partikel}$[d] [nm]
NMC	–	657	0.7	4.9	–
CMC	–	997	1.2	5.0	–
AC	–	617	0.5	3.3	–
Pd/NMC	0.95	625	0.7	4.7	3.4
Pd/CMC	0.98	971	1.2	4.9	4.2
Pd/AC	0.98	473	0.4	3.4	7.8

[a] Pd-Beladung bestimmt durch AAS; [b] Spezifische Oberfläche (BET-Methode); [c] Durchschnittlicher Porendurchmesser (BJH-Methode); [d] Mittlere Partikelgröße aus TEM.

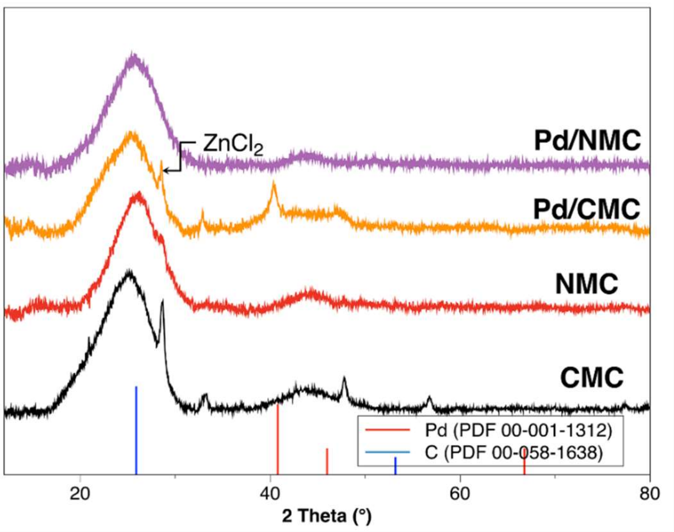

Abbildung S2. Diffraktogramme von Pd/NMC, Pd/CMC und den mesoporösen Kohlenstoffträgern.

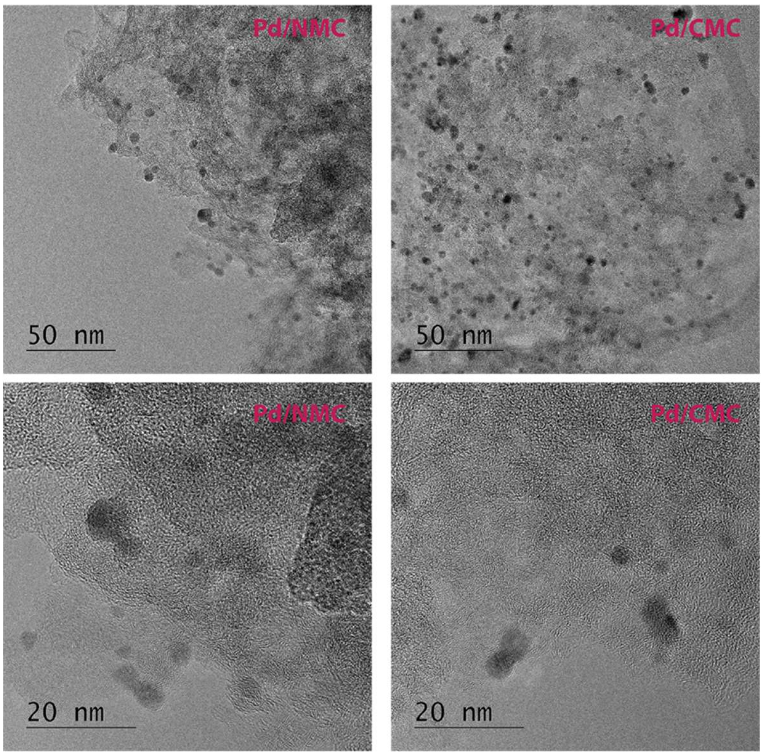

Abbildung S3. TEM-Bilder für Pd/CMC und Pd/NMC nach der Herstellung.

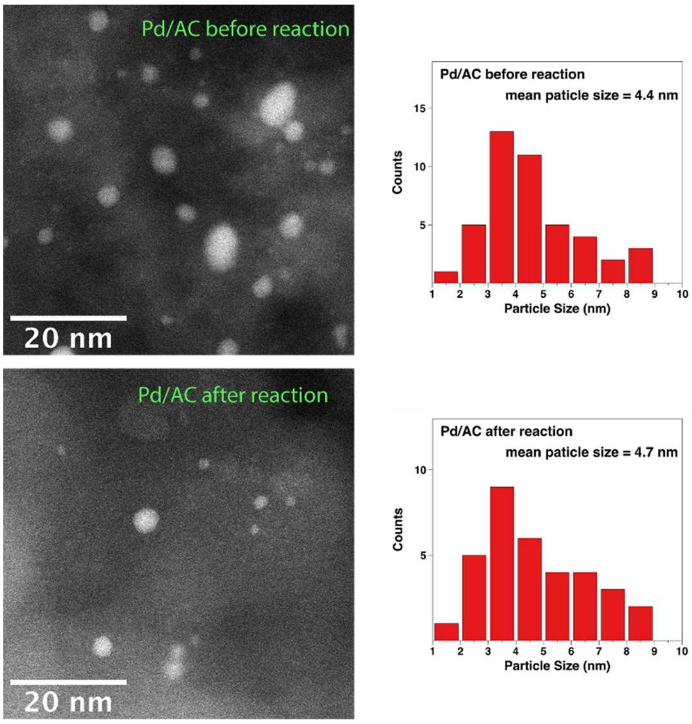

Abbildung S4. HAADF-STEM-Bilder und Pd-Partikelgrößenverteilungen für Pd/AC vor und nach der Reaktion.

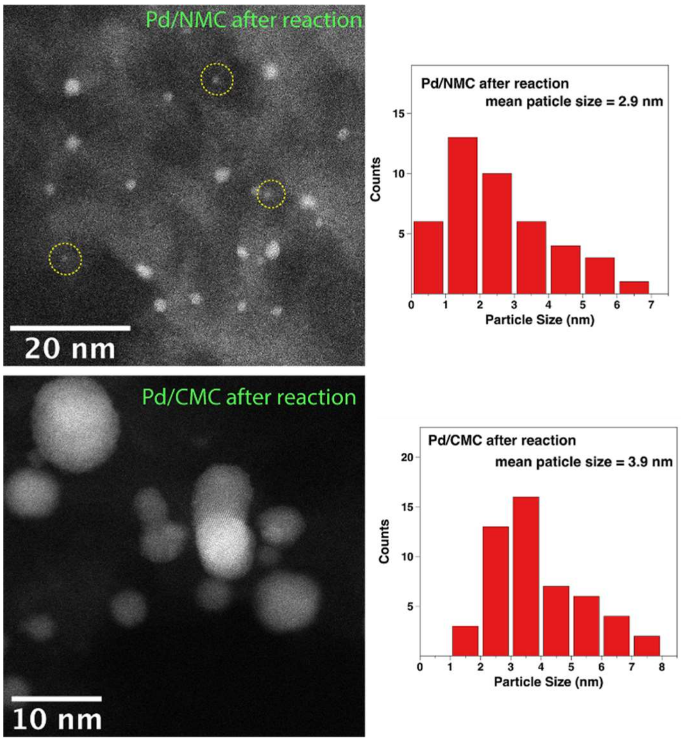

Abbildung S5. HAADF-STEM-Bilder und Pd-Partikelgrößenverteilungen für Pd/NMC und Pd/CMC nach Reaktion in Gegenwart von FA und H_2. Die gelben Kreise beziehen sich auf sub-1 nm Pd NPs oder Cluster.

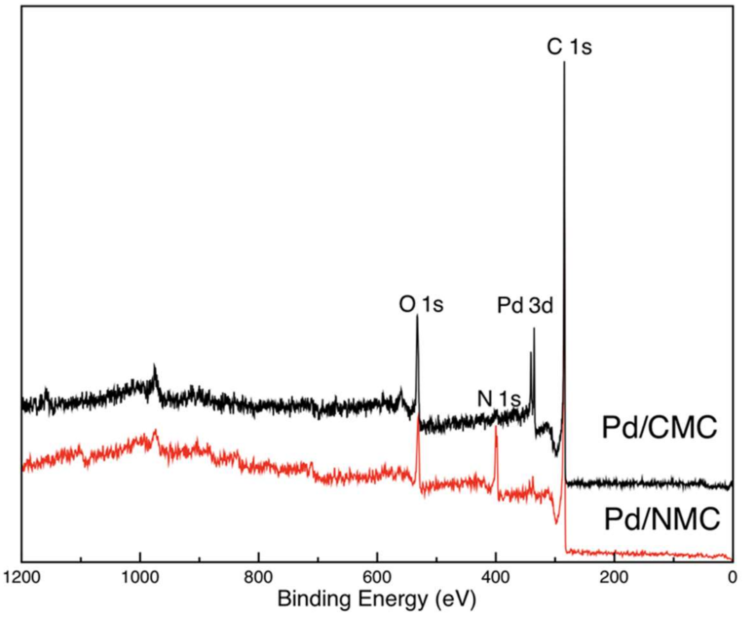

Abbildung S6. XP Übersichtsspektren von Pd/NMC und Pd/CMCnach der Herstellung.

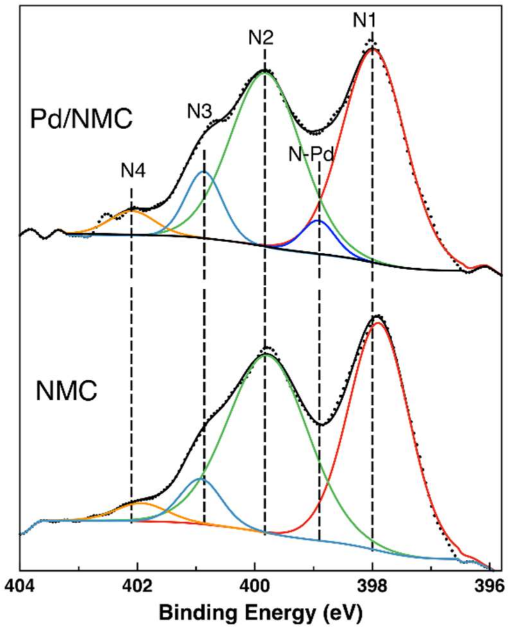

Abbildung S7. N 1s-Bereiche der entfalteten XPS-Ergebnisse für NMC und Pd/NMC nach der Herstellung.[5]

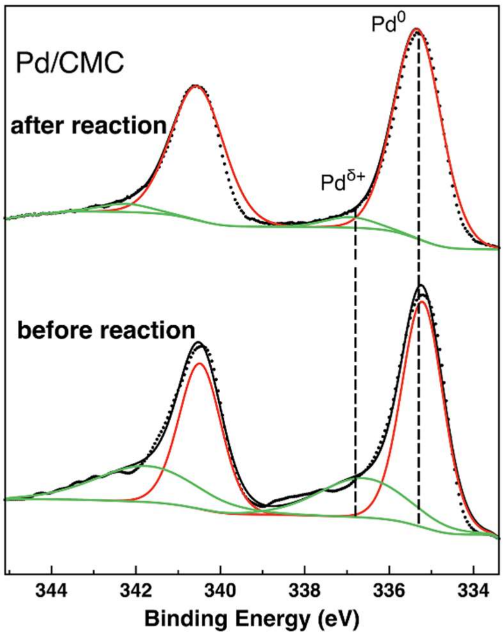

Abbildung S8. Pd 3d-Bereiche der dekonvolutierten XPS-Ergebnisse für Pd/CMC vor und nach der Reaktion in Gegenwart von FA und H_2. Das XPS-Spektrum von Pd/CMC vor der Reaktion wurde in unserem früheren Bericht gezeigt.[5]

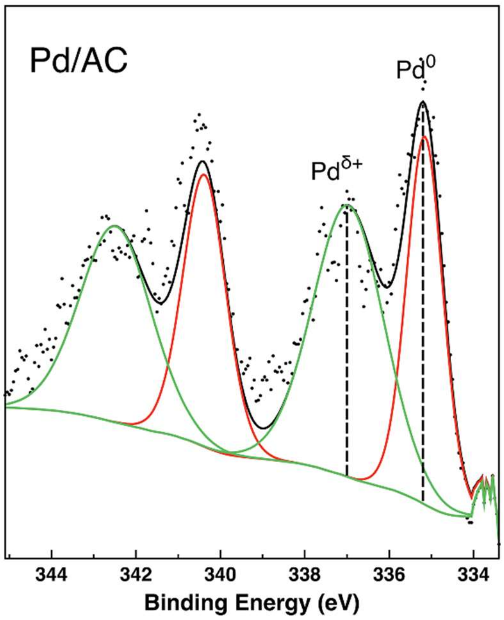

Abbildung S9. Pd 3d-Bereiche der entfalteten XPS-Ergebnisse für Pd/AC nach der Herstellung.

Tabelle S2. Relative Häufigkeit (%) der Komponenten in den Pd $3d_{5/2}$-Spektren

Probe	Komponente/Bindungsenergie (eV)		
	Pd^0 335.0-335.5	$Pd^{\delta+}$ 336.5	Pd^{2+} 337.9
Pd/NMC vor Reaktion	36	14	50
Pd/NMC nach Reaktion	59	11	30
Pd/CMC vor Reaktion*	68	32	–
Pd/CMC nach Reaktion	91	9	–
Pd/AC vor Reaktion	43	57	–

* Die Ergebnisse von Pd/CMC vor der Reaktion wurden in unserem früheren Bericht[5] gezeigt

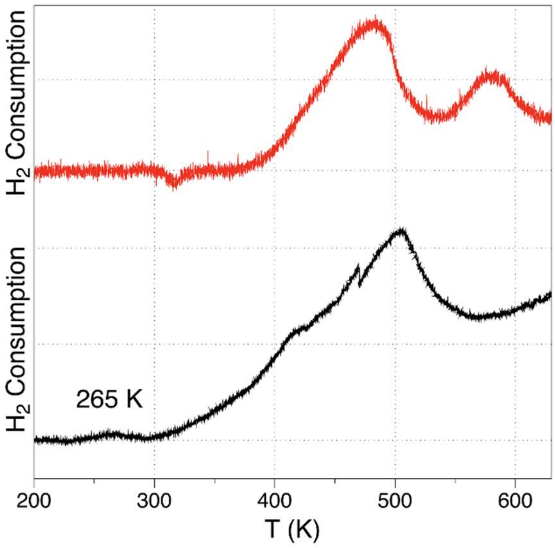

Abbildung S10. H_2 TPR-Profile von Pd/NMC (schwarze Linie) und Pd/CMC (rote Linie) nach der Herstellung.

Die H_2-TPR-Profile von Pd/NMC und Pd/CMC sind in Abbildung S10 dargestellt. Für Pd/NMC wird ein Reduktionspeak bei 265 K beobachtet, der auf die Reduktion von $Pd^{\delta+}$ und Pd^{2+} zu Pd^0 zurückzuführen ist. Im Vergleich dazu wird dieser Reduktionspeak für Pd/CMC nicht beobachtet, wahrscheinlich weil die Pd-NPs in Pd/CMC größtenteils im reduzierten metallischen Zustand vorliegen (siehe Tabelle S1) und die Pd-Beladung gering ist. Die großen

und breiten Peaks mit einer Onset-Temperatur von 300 K für Pd/NMC und 375 K für Pd/CMC sind die Reduktion der funktionellen Oberflächengruppen von NMC bzw. CMC. Es gibt einen negativen Peak bei 315 K, der von der Zersetzung des Palladiumhydrids herrührt. Dieser Palladiumhydrid-Peak wird für Pd/NMC nicht beobachtet, wahrscheinlich aufgrund der Überlappung mit dem breiten Reduktionspeak der funktionellen Oberflächengruppen.

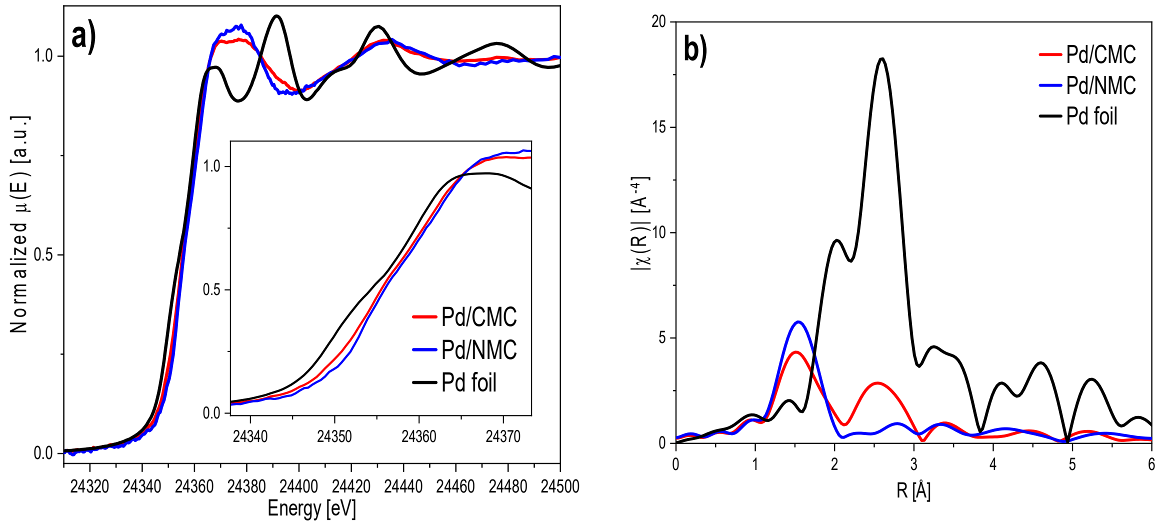

Abbildung S11. XANES (a) und Fourier-transformierte EXAFS-Spektren (b) von präpariertem Pd/CMC und Pd/NMC im Vergleich zu einer Pd-Folie. Es ist zu beachten, dass keine Phasenverschiebungskorrekturen angewendet wurden und die realen Abstände ≈ 0,4 Å größer sein dürften.

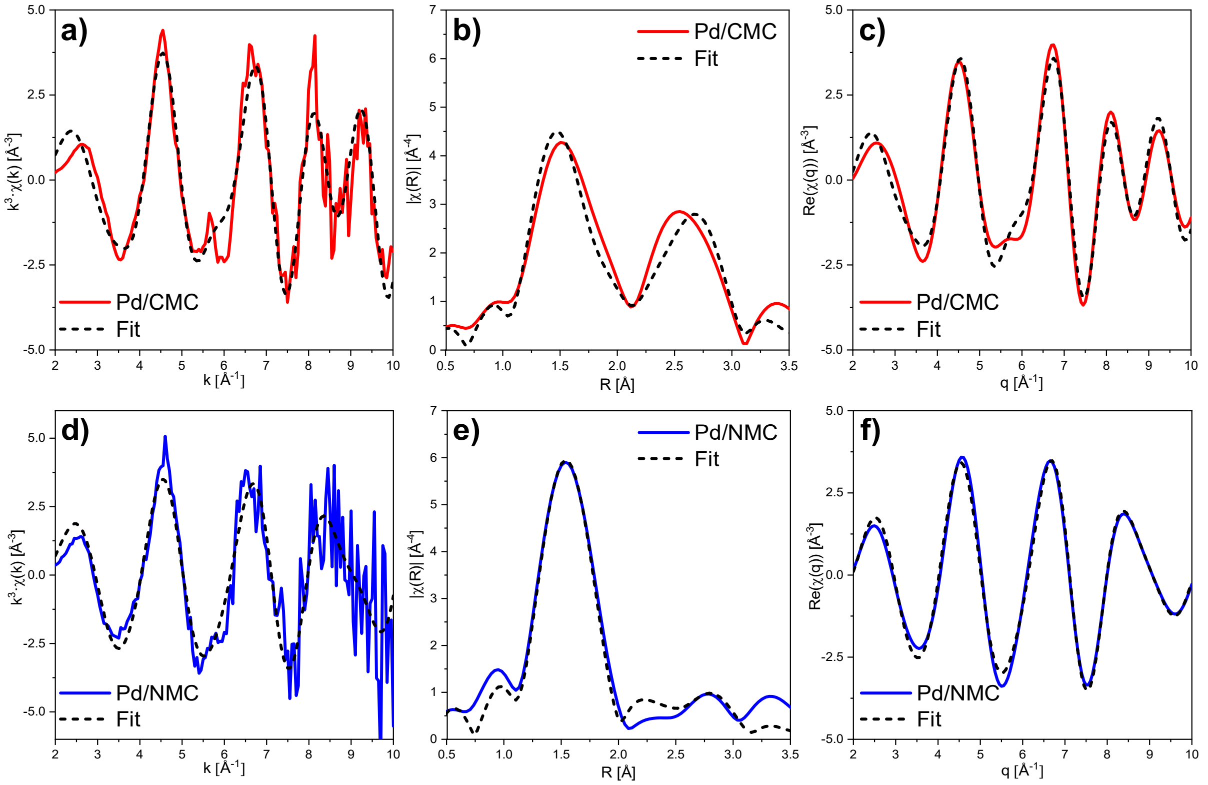

Abbildung S12. Vergleich von experimentell erhaltenen Spektren und den entsprechenden Best-Fit-Berechnungen von wie-hergestellten Pd/CMC (**a** - **c**) und Pd/NMC (**d** - **f**) Materialien. EXAFS-Spektren (**a**, **d**), Fourier-transformierte EXAFS-Spektren (**b**, **e**) und die Rücktransformation davon (**c**, **f**).

Tabelle S3. Beste Anpassungswerte von wie-hergestelltem Pd/CMC und Pd/NMC, erhalten durch EXAFS-Strukturanpassung.

Probe	Abs-Bs[a]	N(Bs)[b]	R(Abs-Bs)[c] [Å]	σ^2 [d] [Å²]
**Pd/CMC**	Pd – O	3.45 ± 0.85	2.02 ± 0.02	0.0062 ± 0.0034
	Pd – Pd	1.96 ± 0.31	2.76 ± 0.03	0.0048*
	S_0^2 [e]= 0.820; ΔE_0 [f] = 6.56 ± 2.56 eV; χ^2_{red}[g] = 17; R[h] = 0.034; N(path)[i] = 2; N(par)[j] = 6; k-Bereich: 2 - 10; R-Bereich: 1 - 3.			
**Pd/NMC**	Pd – N	4.38 ± 0.52	2.06 ± 0.02	0.0031 ± 0.0012
	Pd – Pd	0.80 ± 0.20	2.76 ± 0.03	0.0048*
	S_0^2 [e]= 0.820; ΔE_0 [f] = 7.97 ± 1.34 eV; χ^2_{red}[g] = 1; R[h] = 0.011; N(path)[i] = 2; N(par)[j] = 6; k-Bereich: 2 - 10; R-Bereich: 1 - 3.			

[a] Abs = Röntgenstrahlen absorbierendes Atom, Bs = rückstreuendes Atom. [b] Anzahl der rückstreuenden Atome. [c] Abstand zwischen absorbierendem und rückstreuendem Atom. [d] Debye-Waller-Faktor. [e] Amplitudenreduktionsfaktor. [f] Berücksichtigt die Verschiebung von E_0 zwischen Theorie und Experiment. [g] Reduzierter χ^2 -Fehler (berücksichtigt die Anzahl der unabhängigen Punkte und die Anzahl der variierten Parameter neben dem Fehler zum Experiment). [h] Fit-Index. [i] Gesamtzahl der gefitteten Pfade einschließlich Einzel- und Mehrfachstreupfade. [j] Anzahl der für den Fit verwendeten freien Parameter. * Verwendet als fester Parameter

XAS-Spektren wurden aufgenommen, um den Oxidationszustand zu bestätigen und die lokale chemische Umgebung um die Pd-Zentren zu untersuchen. Die XANES-Spektren beider Proben unterschieden sich deutlich von metallischem Palladium und zeigten eine signifikante Weißlinie, was darauf hindeutet, dass eine größere Menge an Palladiumatomen in einem oxidischen Zustand vorhanden war (Abbildung S11a). Außerdem verschob sich die Kantenposition um etwa 2,5 eV zu höheren Energien (Inset Abbildung S11a), was ebenfalls auf das Vorhandensein von oxidischen Pd-Zentren hindeutet. Eine größere Verschiebung der Kante ($\approx$ 5 eV) wäre jedoch für ein vollständig oxidiertes Material, das nur Pd^{2+} enthält, zu erwarten.[6] Daher enthalten beide Materialien eine Mischung aus $Pd^{2+}/Pd^{\delta+}$ und Pd^0. In Übereinstimmung mit den XPS-Ergebnissen deuten die etwas höhere Intensität der weißen Linie und die etwas größere Verschiebung der Kante darauf hin, dass ein größerer Anteil von Pd in Pd/NMC im Vergleich zu Pd/CMC oxidiert ist.

Für Pd/CMC zeigten die Fourier-transformierten EXAFS-Spektren (Abbildung S11b) das Vorhandensein von zwei Schalen von Rückstreuern bei 1,54 Å und nahe 2,6 Å (es ist zu beachten, dass keine Korrekturen der Phasenverschiebung angewendet wurden und die realen Abstände ≈ 0,4 Å größer sein dürften). Der Vergleich mit der Pd-Folie legt nahe, dass die Schale bei 2,6 Å einer Schale von Pd-Rückstreuern im metallischen Zustand entspricht. Im Gegensatz dazu dürfte die Schale bei 1,54 Å von leichten Rückstreuern wie Kohlenstoff oder Sauerstoff gebildet werden, was für Pd-Spezies, die auf dem Träger verankert sind, zu erwarten wäre. Für Pd/NMC war auch eine Schale bei 1,54 Å aus vermutlich leichten Rückstreuern deutlich vorhanden. Das Vorhandensein einer Schale, die von metallischen Pd-Rückstreuern herrührt, lässt sich jedoch allein durch den Vergleich der Spektren nicht eindeutig bestätigen. Ein Vergleich mit einem Fourier-transformierten EXAFS-Spektrum von PdO[7] bestätigte, dass in beiden Proben keine PdO-Cluster vorhanden waren.

Zusätzlich zur qualitativen Auswertung der Fourier-transformierten EXAFS-Spektren führten wir eine EXAFS-Strukturanpassung durch, um quantitative Informationen über die lokale chemische Umgebung um die Pd-Zentren abzuleiten. Für beide Materialien wurden die besten Anpassungen unter der Annahme von zwei verschiedenen Schalen von Rückstreuungen erhalten (Tabelle S3). Die erste Schale bestand aus leichten Rückstreuern (N, O oder C) und wurde bei 2,02 und 2,06 Å für Pd/CMC bzw. Pd/NMC gefunden. Obwohl wir während der Anpassungsprozedur Sauerstoff- und Stickstoff-Nachbarn für die jeweiligen Materialien angenommen haben, kann diese Technik diese Elemente aufgrund ihrer ähnlichen Rückstreueigenschaften nicht unterscheiden. Die Anzahl der Rückstreuer in der Nähe von 4 entspricht einer quadratisch-planaren Koordinationsgeometrie, die typischerweise für viele

Palladiumkomplexe gefunden wird. Die zweite Schale wurde bei 2,76 Å für beide Materialien gefunden, was mit Pd-Rückstreuern in einem Pd^0-Kristallgitter übereinstimmt. Die Anzahl der Pd-Rückstreuer (1,96 für Pd/CMC, 0,80 für Pd/NMC) ist klein im Vergleich zu einem Pd-Bulk-Kristall (erwartet: 12,0). Es ist zu beachten, dass die geringe Anzahl von Pd-Rückstreuern für Pd/NMC signifikant ist, um eine gute Anpassung zu erhalten. Da die Anzahl der Rückstreuer nur eine Durchschnittszahl ist und stark vom Clusterdurchmesser abhängt,[8] deutet die eher kleine Anzahl von Pd-Rückstreuern darauf hin, dass sehr kleine Pd-Cluster oder sogar isolierte Einzelatome, die eine Koordinationszahl von Null haben, vorhanden sein können. Die geringere Anzahl von Pd-Rückstreuern für Pd/NMC im Vergleich zu Pd/CMC deutet auf einen größeren Anteil von Pd-Clustern oder isolierten Einzelatomen in Pd/NMC als in Pd/CMC hin, was in guter Übereinstimmung mit den STEM-Aufnahmen ist und die Unterschiede in den Pd-Reflexen in deren Diffraktogrammen erklärt. Darüber hinaus war eine Größenbestimmung auf Basis der EXAFS-Strukturanpassung nicht sinnvoll, da nicht völlig ausgeschlossen werden kann, dass einige isolierte Pd^{2+}-Einzelatomspezies im Material vorhanden sind, was die Größenbestimmung negativ beeinflussen würde. Nichtsdestotrotz unterstützen die geringe Anzahl von Rückstreuern und die geringe Intensität der Schale bei 2,6 Å in den Fourier-transformierten EXAFS-Spektren die Annahme von kleinen Pd-Clustern im unteren Nanometerbereich.

Katalytische Ergebnisse

Tabelle S4. Katalytische Aktivität von verschiedenen Katalysatoren bei der Hydrogenolyse von HMF zu DMF.

Eintrag	Katalysator	Reaktand	Wasserstoff Quelle	Zeit (h)	T (°C)	Lösungs mittel	Umsatz (mol %)	S_{DMF} (mol %)	TOF (h^{-1})	Ref.
1	Pd/NMC	HMF	FA+H_2	2	160	THF	> 99.9	> 97.0	150	T[a]
2	Pd/NMC	HMF	FA	3	160	THF	60.8	64.3	41	T[a]
3	Pd/CMC	HMF	FA+H_2	3	160	THF	90.1	80.0	75	T[a]
4	Pd/CMC	HMF	FA	3	160	THF	19.4	62.9	13	T[a]
5	Pd/C	FMF	FA	15	120	Dioxan	> 95.0	> 95.0	1.2×10^{-3}	9
6	Pd/C	FMF	FA+H_2	15	120	Dioxan	> 95.0	87.6	0.27×10^{-3}	9
7	Pd/C/Zn	HMF	H_2	8	150	THF	> 99.9	85.0	27.2	10
8	Ni-Co/C	HMF	FA	24	210	THF	> 99.0	90.0	0.1	11
9	Ru/MoO_x/C	HMF	H_2	1	180	n-Butyl alkohol	> 99.0	79.8	21.3	12

[a] Diese Arbeit, Reaktionsbedingungen: 1,5 mmol HMF, 50 mg Katalysatoren, 30 mL THF, 160 ℃, 2 oder 3 h;

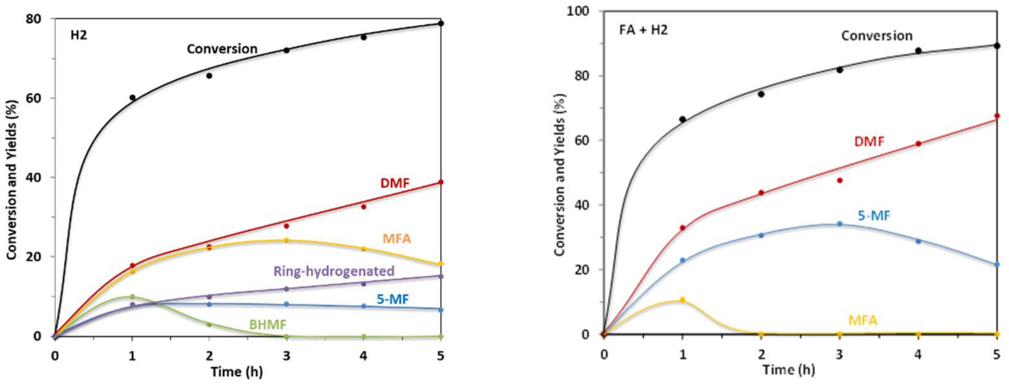

Abbildung S13. Zeitlicher Verlauf der Hydrogenolyse von HMF zu DMF über Pd/CMC mit H_2 und mit FA + H_2.

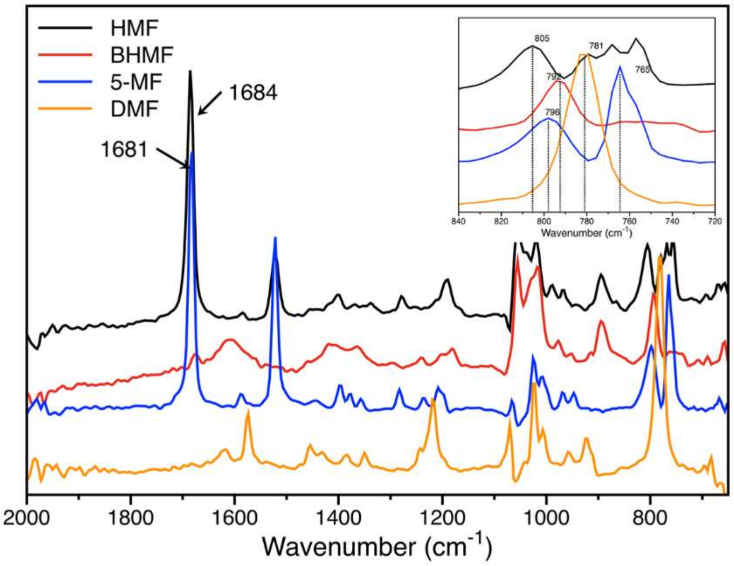

Abbildung S14. ATR-IR Spektren von Standardproben bei 0,1 M in THF.

Tabelle S5. ATR-IR-Absorptionsbanden der Standardproben in THF.

	**ν(C=O) (cm^{-1})**	**ν(C=C) (cm^{-1})**	**ν(C−H) (cm^{-1})**
**HMF**	**1684**	**1520**	**805,779,767,756**
**BHMF**	**-**	**1608**	**792**
**5-MF**	**1681**	**1520**	**796,765**
**DMF**	**-**	**1573**	**781**

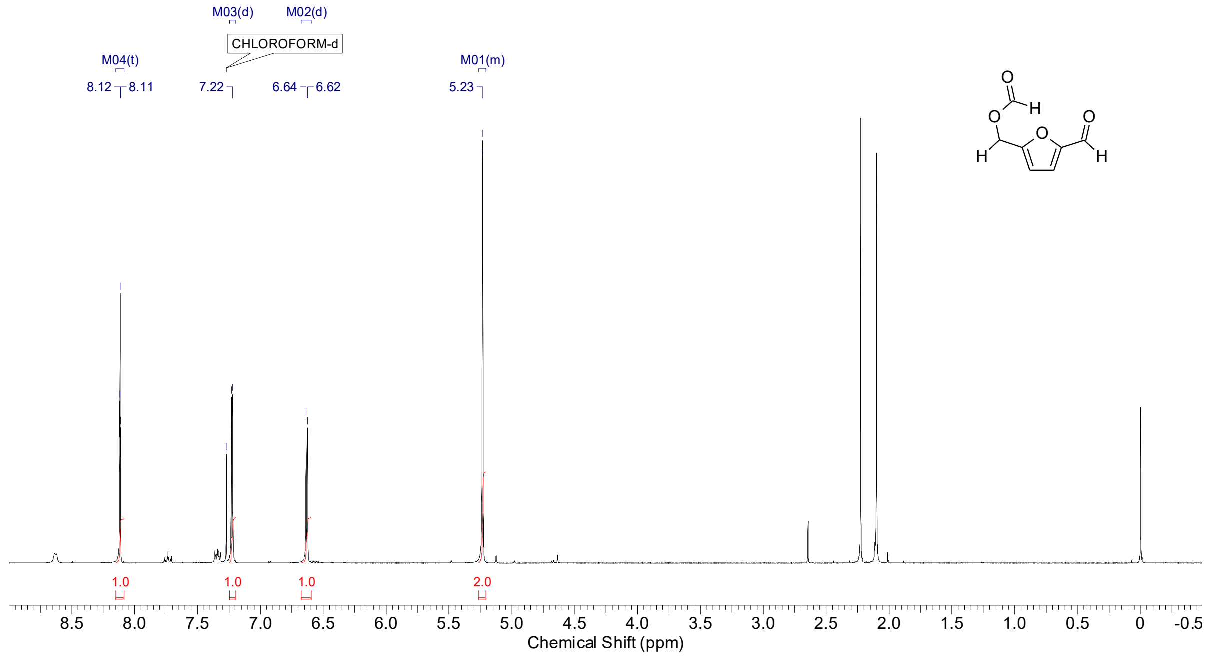

Abbildung S15. ^{1}H NMR Spektrum of FMF, ^{1}H NMR (300 MHz, *CHLOROFORM-d*) δ ppm 8.65 (s, 1 H), 8.12 (t, *J*=0.9 Hz, 1 H), 7.22 (d, *J*=3.6 Hz, 1 H), 6.63 (d, *J*=3.5 Hz, 1 H), 5.21 - 5.26 (m, 2 H).

Berechnungen

Methodik

Die quantenchemische Untersuchung wurde unter Verwendung der Dichtefunktionaltheorie (DFT) mit dem TPSS[13] -Funktional und dem def2-SVP[14] -Basissatz durchgeführt, der Stuttgart-Köln-effektive Kernpotentiale (def2-ecp)[15] für die Pd-Atome enthält. Alle Berechnungen wurden unter Verwendung des Programmpakets TURBOMOLE[16] mit der Multipole Accelerated Resolution of Identity (MARI-J)[17] -Näherung mit optimierten Hilfs-Basissätzen[18] und Grimme's D3[19] -Korrektur für Londoner Dispersionswechselwirkungen durchgeführt.

Das Modell von Pd/CMC, ein Pd_{21}-Cluster auf einer wasserstoffterminierten Graphenschicht aus 150 Kohlenstoffatomen, hat sich in einer früheren Studie als vernünftige Wahl erwiesen.[20] Für das bifunktionelle Pd/NMC-System wurden drei Kohlenstoffatome des Pd/CMC-Systems durch Stickstoffatome ersetzt. Die stabilsten Strukturen dieser Modelle sowie die Minima von Ameisensäure und Wasserstoff an diesen Systemen wurden durch Grundzustandsgeometrie-Optimierungen bestimmt. Für die relativen Energien wurden die Nullpunktsschwingungsenergie-Korrekturen (ZPVE) aus der Schwingungsanalyse einbezogen. Da die potentiellen Energieflächen der Pd/CMC- und Pd/NMC-Systeme oft sehr flach sind, zeigt die Schwingungsanalyse einiger Systeme noch eine (sehr kleine) negative Frequenz. Wir erwarten, dass die dadurch verursachten Fehler vernachlässigbar sind. Für die Bestimmung der Reaktionswege und das Auffinden initialer Übergangszustandsstrukturen wurde die im Wölflingsmodul von TURBOMOLE[21] implementierte Chain-of-State-Methode verwendet. Die anfänglichen Übergangszustandsstrukturen wurden dann mit dem eigenwertfolgenden TRIM-Algorithmus (trust-region image minimization)[22] weiter optimiert und durch IRC-Berechnungen (intrinsic reaction coordinate)[23] validiert. Adsorptions- und Dissoziationsenergien sind mit einem negativen Vorzeichen versehen, während Energiebarrieren wie Aktivierungsbarrieren ein positives Vorzeichen haben. Atomare Ladungen wurden mit Hilfe von natürlichen Populationsanalysen (NPAs) bestimmt.[24]

Tabelle S6. Adsorptionsenergien von FA an Pd_{21}.

Modelsystem	FA Konfiguration	ΔE_{ads} / kJ mol^{-1}
**Pd_{21}/CMC**	**C=O Bindung**	**-129.6**
**Pd_{21}/CMC**	**C Atombindung**	**-75.5**
**Pd_{21}/NMC**	**C=O Bindung**	**-132.5**
**Pd_{21}/NMC**	**C Atombindung**	**nicht stabil[a]**

[a] Für die C-Atombindung an Pd_{21}/NMC wurden nur Konfigurationen mit imaginären Frequenzen gefunden.

Tabelle S7. Dissoziationsenergien von H_2 an Pd_{21} in kJ mol^{-1}.

Model system	H Atom konfiguration	TPSS/ def2-SVP	TPSS/ def2-TZVP	PBE/ def2-SVP
**Pd_{21}/CMC**	**2 H in fcc Position**	**-111.1**	**-94.9**	**-122.0**
**Pd_{21}/CMC**	**1 H in fcc, 1 H am Rand**	**-96.0**	**-86.0**	**-116.8**
**Pd_{21}/NMC**	**2 H in fcc Position**	**-118.9**	**-112.4**	**-131.2**
**Pd_{21}/NMC**	**1 H in fcc, 1 H am Rand (nahe Pd^{2+})**	**-116.9**	**-111.2**	**-129.1**
**Pd_{21}/NMC**	**1 H in fcc, 1 H am Rand (fern von Pd^{2+})**	**-112.6**	**-108.2**	**-126.1**

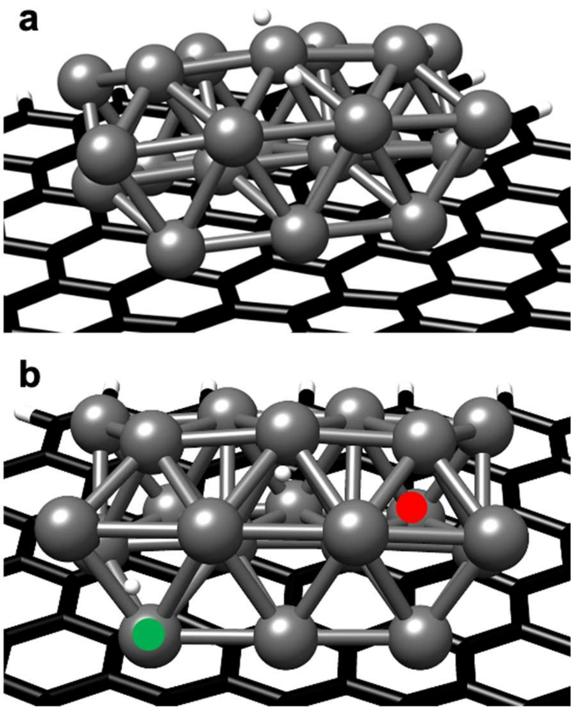

Abbildung S16. H-Atomkonfigurationen auf Pd_{21}/CMC: (a) 2 H in fcc-Positionen; (b) 1 H in fcc, 1 H am Rand. Für Pd_{21}/NMC: unteres linkes Pd-Atom (grün; nah) oder hinteres rechtes Pd-Atom (rot; fern) ist mit 2 pyridinischen N-Atomen verbunden.

Referenzen

1. Y. A. Huang, S. Hu, S. Zuo, Z. Xu, C. Han, J. Shen, *J. Mater. Chem.* **2009**, *19*, 7759-7764.
2. Y. A. Huang, F. Yang, Z. Xu, J. Shen, *J. Colloid Interf. Sci* **2011**, *363*, 193-198.
3. E. Welter, R. Chernikov, M. Herrmann, R. Nemausat, *AIP Conf. Proc.* **2019**, *2054*, 04002.
4. B. Ravel, M. Newville, *J. Synchrotron Rad.* **2005**, *12*, 537-541
5. L. Warczinski, B. Hu, T. Eckhard, B. Peng, M. Muhler, C. Hättig, *Phys. Chem. Chem. Phys.* **2020**, *https://doi.org/10.1039/D0CP03234D*.
6. K. A. Karinshak, P. Lott, M. P. Harold, O. Deutschmann, *ChemCatChem* **2020**, *12*, 3712-3720.
7. K. Okumura, J. Amano, N. Yasunobu, M. Niwa, *J. Phys. Chem. B* **2000**, *104*, 1050–1057.
8. A. Jentys, *Phys. Chem. Chem. Phys.* **1999**, *1*, 4059-4063
9. J. Mitra, X. Zhou, T. Rauchfuss, *Green Chem.* **2015,** *17*, 307-313.
10. B. Saha, C. M. Bohn, M. M. Abu-Omar, *ChemSusChem* **2014,** *7*, 3095-3101.
11. P. Yang, Q. Xia, X. Liu, Y. Wang, *Fuel* **2017,** *187*, 159-166.
12. Y. Yang, Q. Liu, D. Li, J. Tan, Q. Zhang, C. Wang, L. Ma, *RSC Advances* **2017,** *7*, 16311-16318.
13. J. Tao, J. P. Perdew, V. N. Staroverov, G. E. Scuseria, *Phys. Rev. Lett.* **2003,** *91* (14), 146401.
14. F. Weigend, R. Ahlrichs, *Phys. Chem. Chem. Phys.* **2005,** *7* (18), 3297-3305.
15. D. Andrae, U. Häußermann, M. Dolg, H. Stoll, H. Preuß, *Theor. Chim. Acta* **1990,** *77* (2), 123-141.
16. Turbomole V7.3 2018, a development of University of Karlsruhe and Forschungszentrum Karlsruhe GmbH, 1989-2007, TURBOMOLE GmbH, since 2007, available from http://www.turbomole.com.
17. M. Sierka, A. Hogekamp, R. Ahlrichs, R., *J. Chem. Phys.* **2003,** *118* (20), 9136-9148.
18. F. Weigend, *Phys. Chem. Chem. Phys.* **2006,** *8* (9), 1057-1065.
19. S. Grimme, J. Antony, S. Ehrlich, H. Krieg, *J. Chem. Phys.* **2010,** *132* (15), 154104.
20. L. Warczinski, C. Hättig, *Phys. Chem. Chem. Phys.* **2019,** *21* (38), 21577-21587.
21. P. Plessow, *J. Chem. Theory Comput.* **2013,** *9* (3), 1305-1310.
22. T. Helgaker, *Chem. Phys. Lett.* **1991,** *182* (5), 503-510.
23. K. Fukui, *Acc. Chem. Res.* **1981,** *14* (12), 363-368.
24. A. E. Reed, R. B. Weinstock, F. Weinhold, *J. Chem. Phys.* **1985,** *83* (2), 735-746.
